# Illusion of knowledge in statistics among clinicians: evaluating the alignment between objective accuracy and subjective confidence, an online survey

**DOI:** 10.1186/s41235-023-00474-1

**Published:** 2023-04-20

**Authors:** Camille Lakhlifi, François-Xavier Lejeune, Marion Rouault, Mehdi Khamassi, Benjamin Rohaut

**Affiliations:** 1 Sorbonne Université, Institut du Cerveau - Paris Brain Institute - ICM, Inserm, CNRS, APHP, Hôpital de la Pitié Salpêtrière, Paris, France; 2 Université Paris Cité, Paris, France; 3 Hôpital de la Pitié Salpêtrière, Paris Brain Institute’s Data Analysis Core, Paris, France; 4 Département d’Études Cognitives, École Normale Supérieure, Université Paris Sciences & Lettres (PSL University), Paris, France; 5 Institute of Intelligent Systems and Robotics, CNRS, Sorbonne Université, Paris, France; 6 AP-HP, Hôpital de la Pitié Salpêtrière, DMU Neurosciences, Paris, France

**Keywords:** Statistical illiteracy, Metacognition, Overconfidence bias, Sensitivity, Calibration, Discrimination, Decision-making, Medical context, Conditional probabilities, Natural frequencies

## Abstract

Healthcare professionals’ statistical illiteracy can impair medical decision quality and compromise patient safety. Previous studies have documented clinicians’ insufficient proficiency in statistics and a tendency in overconfidence. However, an underexplored aspect is clinicians’ awareness of their lack of statistical knowledge that precludes any corrective intervention attempt. Here, we investigated physicians’, residents’ and medical students’ alignment between subjective confidence judgments and objective accuracy in basic medical statistics. We also examined how gender, profile of experience and practice of research activity affect this alignment, and the influence of problem framing (conditional probabilities, CP vs. natural frequencies, NF). Eight hundred ninety-eight clinicians completed an online survey assessing skill and confidence on three topics: vaccine efficacy, *p* value and diagnostic test results interpretation. Results evidenced an overall consistent poor proficiency in statistics often combined with high confidence, even in incorrect answers. We also demonstrate that despite overconfidence bias, clinicians show a degree of metacognitive sensitivity, as their confidence judgments discriminate between their correct and incorrect answers. Finally, we confirm the positive impact of the more intuitive NF framing on accuracy. Together, our results pave the way for the development of teaching recommendations and pedagogical interventions such as promoting metacognition on basic knowledge and statistical reasoning as well as the use of NF to tackle statistical illiteracy in the medical context.

**Supplementary Information:**

The online version contains supplementary material available at 10.1186/s41235-023-00474-1.

## Introduction

### Study background

#### Statistical illiteracy among clinicians

In medicine, a substantial amount of decisions is made relying on statistical and numerical information. To make a diagnosis, prescribe a treatment or a medical procedure, evaluate a prognosis, clinicians have to reason and decide based on parameters such as disease prevalence, test results, cost/benefit ratio of each intervention, etc. Previous studies have suggested that an insufficient proficiency in basic statistics can not only be observed in the general public, but also in professionals with relevant expertise, including clinicians (Gigerenzer & Gray, 2013; Gigerenzer et al., 2007). This statistical illiteracy can impact medical practice and have serious consequences (such as overdiagnosis or overtreatment), or even compromise patient safety by altering the quality of clinical decisions (Gaissmaier & Gigerenzer, 2008; Gigerenzer et al., 2007; Jenny et al., 2018; Wegwarth, 2013; Wegwarth & Gigerenzer, 2013; Wegwarth et al., 2012).

#### Diagnosis test results’ interpretation

One specific medical task on which a lack of statistical literacy has been described consists of interpreting a diagnosis test result by estimating its associated positive/negative predictive value (PPV/NPV). The PPV represents the likelihood for a patient receiving a positive test result to actually have the disease. PPV is calculated based on the test’s performance values (namely: sensitivity and specificity) and the disease prevalence (also known as the pre-test probability). Hoffrage and Gigerenzer (1998) previously showed that when the relevant information to perform the PPV calculation is given in conditional probabilities (standard statistical problem framing in the medical context (Altman & Bland, 1994a, 1994b)), clinicians often confuse PPV with sensitivity and only get the correct answer in 10% of the cases (Gigerenzer et al., 2007; Hoffrage & Gigerenzer, 1998). Several other studies confirmed clinicians’ difficulties while facing this PPV calculation task (Anderson et al., 2014; Bramwell et al., 2006; Casscells et al., 2010; Eddy, 1982; Hoffrage et al., 2000; Labarge et al., 2003; Lindsey et al., 2002; Young et al., 2002). This type of exercise could be seen as a cognitive reflection test for clinicians and provides a good testbed to assess health care practitioners' statistical (il)literacy.

#### Proposed solutions

Recommendations to tackle statistical illiteracy can be of two types: top-down, by teaching people how to better understand statistical information and promote metacognition, or bottom-up by adapting the environment and especially the way we provide numbers to facilitate their processing (Grüne-Yanoff & Hertwig, 2016). Making the information more digestible could enable both practitioners and patients to make better-informed decisions, thus empowering decision-makers (Grüne-Yanoff & Hertwig, 2016). Several insights could improve our communication of numbers, mainly relying on choosing intuitive ways of framing statistical information: prioritize frequency statements instead of single-event probabilities, absolute over relative risks (Gigerenzer et al., 2007), mortality rates instead of survival rates, graphical representations over numerical information (Kurz-Milcke et al., 2008), and natural frequencies (NF) over conditional probabilities (CP). PPV calculation is often taught early in the medical curriculum using conditional probabilities (Bayes theorem). As such, the statistical parameters provided to perform the PPV calculation for a given test are usually expressed using percentages (example: “the prevalence of the disease D in the population of interest is 1%, and the sensitivity and specificity of the test are 90% and 91%, respectively”). Alternatively, natural frequencies (NF) are suggested to allow a more simple and intuitive display of the same information (Gigerenzer & Hoffrage, 1995; Gigerenzer et al., 2007). NF represent absolute numerical information that one could empirically and sequentially observe in a population and also provide clearly the reference class (Gigerenzer & Edwards, 2003; Gigerenzer & Hoffrage, 1995) (example: “in the population of interest, 10 persons out of 1 000 have the disease D; among the 10 sick persons, 9 will receive a positive test result while 901 out of the 990 healthy persons will receive a negative test result”). In this example, the PPV appears intuitively [9/(9 + 89)] and does not need complex calculation. It has been shown that teaching PPV calculation using NF positively impacts the accuracy of healthcare professionals at estimating PPV when compared to CP (46% vs. 10%, respectively; Hoffrage & Gigerenzer, 1998). More recently, after obtaining strong cues of a poor statistical literacy of medical students and residents through a 10-item survey, an interventional experiment concluded for a positive impact of a 90-min training session on risk literacy based on NF (rather than CP) to explain sensitivity and specificity (Jenny et al., 2018), replicating previous similar results (Sedlmeier & Gigerenzer, 2001). These findings suggest that errors in statistical reasoning are not stable and can be addressed with proper educational alternatives: as such, NF could be a simple and effective tool to minimize confusions and errors arising from CP (Gigerenzer & Edwards, 2003).

#### Metacognition, confidence & (mis)calibration in clinical decisions

Another approach which could help mitigate clinicians’ error in statistical reasoning would be to promote reflexive thinking and to increase their awareness of their actual statistical performance. This ability to assess, reflect on and monitor one’s own thoughts and decisions is usually referred to as metacognition (Fleming & Lau, 2014; Norman et al., 2019). The most common way to measure metacognition in laboratory-based experiments is to collect confidence judgments (subjective assessment of whether their decision is right or not) (Rouault et al., 2022), and two aspects have been traditionally studied. First, metacognitive bias refers to our general tendency to report a high/low confidence. Second, metacognitive sensitivity refers to our capacity to discriminate between our correct and incorrect decisions (Fleming et al., 2012). At the level of the general population, most of us are somewhat overconfident (Moore & Healy, 2008; Rouault et al., 2018), but can detect and recognize our errors (Yeung & Summerfield, 2012). The accuracy of our confidence judgments could in turn impact our decision-making process by engaging in seeking more information, a second opinion, or exploring alternative options for instance. As such, metacognition is often considered as a potential solution to address the prevalence and impact of cognitive biases such as overconfidence in clinical context (Croskerry, 2013; Hershberger et al., 1995; Reilly et al., 2013). A relevant alignment of confidence on accuracy stands as a key parameter in bridging the gap between uncertainty, confidence and clinical challenges (Zwaan & Hautz, 2019). Indeed, clinicians sometimes seem unable to accurately self-assess their fitness-to-perform (Davis et al., 2006; Huizinga et al., 2019) and more likely than other professionals to underestimate the impact of stress and fatigue on their own practice, and their risks of committing errors (Klein, 2005; Sexton et al., 2000).

Overconfidence is one of the three most studied and reported cognitive biases identified in the clinical context (Saposnik et al., 2016). It is typically studied using a standard experimental paradigm that first measures accuracy with one or several real-life exercises or a survey with clinical vignettes (e.g., diagnosis cases) with a specific degree of difficulty that participants are asked to solve. Second, their confidence in the accuracy of their answers is collected (most of the time retrospectively), often by asking explicitly the participants to report after each vignette how confident they are that they correctly solved the task (found the right answer, performed well, made the optimal decision…) (Olsson, 2014). The comparison between their confidence (second-order judgments about their accuracy) and their actual accuracy (first-order task performance) for all responses is used to determine their calibration (Fleming & Lau, 2014; Rahnev et al., 2020). Calibration therefore reflects how well confidence tracks accuracy overall. The conclusions of such studies often highlight miscalibration, most of the time toward overconfidence, among medical students and clinicians (Ahmed & Walsh, 2020; Barnsley et al., 2004; Berner & Graber, 2008; Borracci & Arribalzaga, 2018; Brezis et al., 2019; Davis et al., 2006; Friedman et al., 2001, 2005; Graber, 2008; Klein, 2005; Lam & Edward Feller, 2020; Lawton et al., 2019; Meyer et al., 2013; Miller et al., 2019; Morgan & Cleave-Hogg, 2002; Naguib et al., 2019; O’Donoghue et al., 2018; Rahmani, 2020; Zwaan & Hautz, 2019). However, metacognitive sensitivity (how well confidence judgments discriminate between correct and incorrect answers) has barely been addressed in these previous works (Barnsley et al., 2004; Fleming & Lau, 2014; C. Friedman et al., 2001; C. P. Friedman et al., 2005; Maniscalco & Lau, 2012; Meyer et al., 2013; O’Donoghue et al., 2018).

### Rationale and objectives of the present study

While previous studies suggest a poor proficiency in statistics and a tendency to overconfidence among medical students and clinicians, an underexplored but crucial aspect is doctors’ awareness of their lack of knowledge in statistical reasoning. Despite the numerous studies conducted on statistical illiteracy throughout the past decades, to our knowledge, only one explicitly investigated clinicians’ confidence in their statistical knowledge (Bramwell et al., 2006). This work revealed a miscalibration toward overconfidence (Bramwell et al., 2006). However, this study only collected low-granularity confidence judgments with Likert scales, solely focused on a PPV calculation task, and did not explore how clinicians’ demographics nor statistical problem framing affected reported confidence. Furthermore, it remains unknown how well reported confidence distinguishes between objectively correct and incorrect responses. Hence, the present study aims at addressing these gaps. We used a paradigm inspired by previous studies (Bramwell et al., 2006; Hoffrage & Gigerenzer, 1998; Jenny et al., 2018), collecting both accuracy and confidence judgments in a large sample of medical students and clinicians (*N* = 898) on three different topics related to medical statistics: vaccine efficacy, *p* value and test results interpretation. Our results indicate a low response accuracy across these three topics, and provide evidence that despite an overconfidence bias, clinicians still showed a substantial degree of metacognitive sensitivity (measured via discrimination).

## Methods

### Survey content

**Fig. 1:**
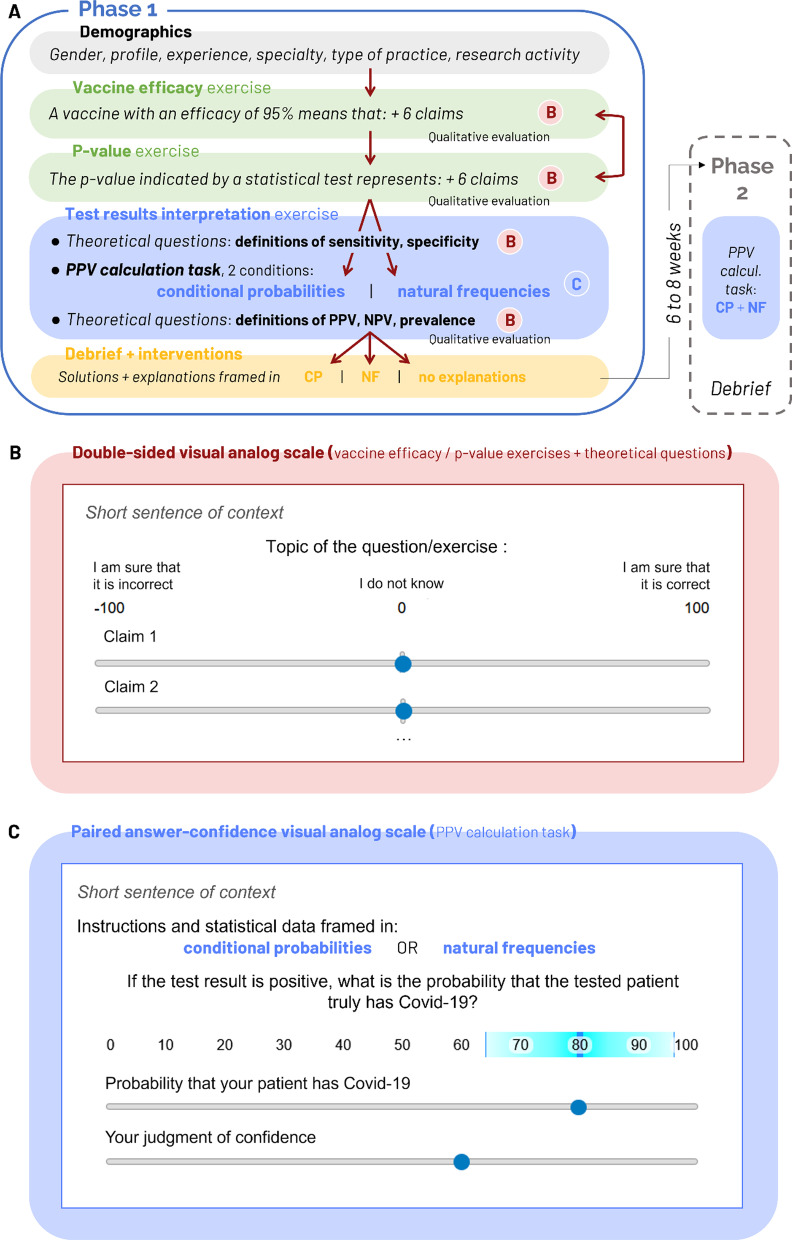
Experimental paradigm and measurements scales. **A** Study design. Exercises focusing on vaccine efficacy and *p* value were presented in a random order, while the exercise about test results interpretation was always the last. In this figure section, the letters B and C, respectively, refer to sections B and C from Fig. 1, each section describing the response interface used for some questions. The full survey content including sentences of context, questions as well as explanations is provided in *Annex* of Additional file 1. **B** A double-sided visual analog scale was used in exercises about vaccine efficacy and *p* value as well as in the five theoretical questions of the exercise about test results interpretation to collect simultaneously participants’ accuracy (side of the cursor) and confidence (distance to the center/extremity of the scale) on each claim. *Instructions given to the participants* To give your answer, move the slider on the scale: the more hesitant you are about your answer, the closer the slider should be placed to the middle, the more confident you are about your answer, the closer the slider should be placed to the extremity of the scale. If you don't know and you answer randomly, place the slider on the center. **C** The practical PPV calculation task of the exercise focused on test results interpretation used two paired visual analog scales: participants indicated their PPV estimation (ranging from 0 to 100%) by moving the first cursor on the first visual analog scale, and then reported their judgment of confidence by adjusting the width of a blue zone of uncertainty around the first value using the second cursor of the second visual analog scale (width of blue zone = 0.02 * confidence^2^). The lower the judgment of confidence, the more extended the blue area. *Instructions given to the participants* To answer, move the first slider on the scale to indicate your answer, then adjust your confidence level by moving the second slider

This study was performed using a preregistered online survey framed as a challenging quiz constituted of three exercises. Participants were provided with broad explanations regarding the quiz content as well as instructions and examples on how to answer using visual analog scales (VAS). Participants were asked not to use any external help (Internet, book or colleague) to answer the quiz. The survey structure is explained in Fig. 1A; its content including sentences of context, questions as well as explanations is provided in *Annex* of Additional file 1.

#### Demographics

In addition to the total completion time, the following demographic data were collected: gender, age, profile (student, resident, physician), level of experience (number of years of studies/residency/practice), involvement in studying another discipline (especially mathematics), country of study/practice, intended (for students) or practiced medical specialty, type of practice (public/private, hospital/medical office) and relative time shared between clinical practice and research activities.

After collecting demographics, participants were presented with the three exercises collecting both answers and confidence judgments for each question. Each exercise focused on a specific statistical topic (vaccine efficacy, *p* value and test results interpretation). Vaccine efficacy and test results interpretation was applied to the current context of the COVID-19 pandemic.

#### Exercises 1 and 2: vaccine efficacy and *p* value exercises

**Fig. 2:**
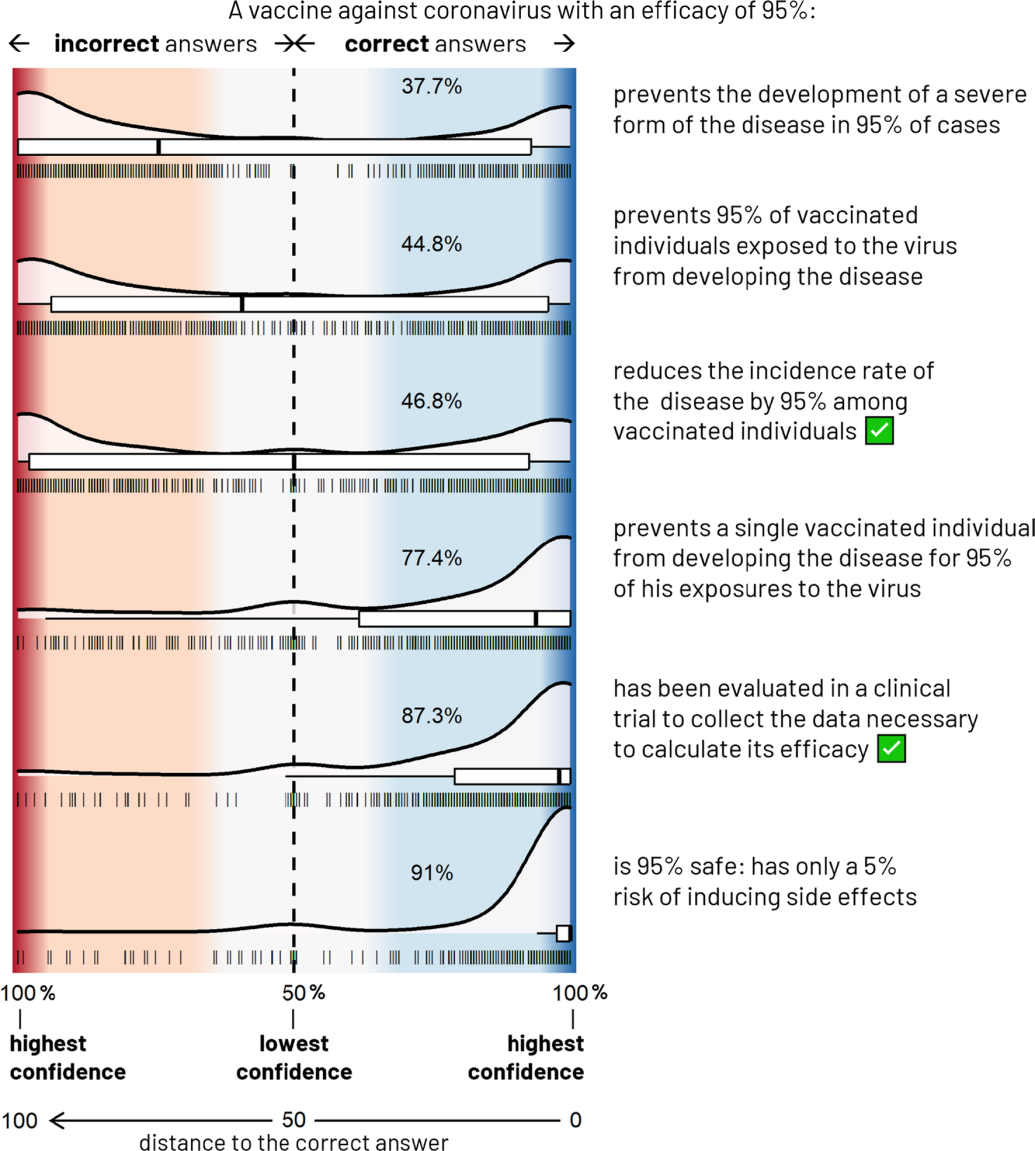
Responses to the “vaccine efficacy” exercise, N = 822. The density and box plots represent the distribution of participants’ responses for each of the 6 proposed claims. The collected data are here mapped onto a double-sided probability scale ranging from 50% (lowest confidence, the participant answered randomly) to 100% (maximal confidence judgment), both for correct (blue area) and incorrect (red area) answers. Also represented on the x-axis, the distance to the correct answer (d) is defined as a score composed both by the accuracy and confidence, ranging from 0: correct answer with maximal confidence to 100: incorrect answer with maximal confidence through 50: “I do not know”, d < 50 and d > 50, respectively, corresponding to correct and incorrect answers. Each vertical line stands for a response. The percentage of this exercise’s participants who gave a correct answer is indicated in each blue area. True claims are indicated by a green ticked box

**Fig. 3:**
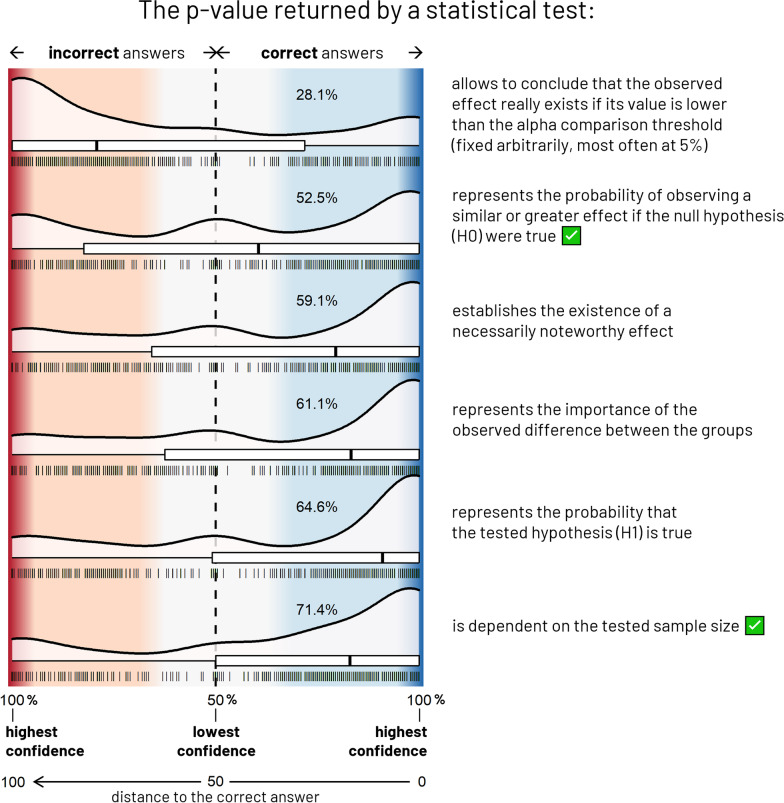
Responses to the “*p* value” exercise, N = 794. The density and box plots represent the distribution of participants’ responses for each of the 6 proposed claims. The collected data are here mapped onto a double-sided probability scale ranging from 50% (lowest confidence, the participant answered randomly) to 100% (maximal confidence judgment), both for correct (blue area) and incorrect (red area) answers. Also represented on the x-axis, the distance to the correct answer (d) is defined as a score composed both by the accuracy and confidence, ranging from 0: correct answer with maximal confidence to 100: incorrect answer with maximal confidence through 50: “I do not know”, d < 50 and d > 50, respectively, corresponding to correct and incorrect answers. Each vertical line stands for a response. The percentage of this exercise’s participants who gave a correct answer is indicated in each blue area. True claims are indicated by a green ticked box

The two first exercises were presented in a random order, and focused on the topic of “vaccine efficacy” and “*p* value”, respectively. Each was composed of a short sentence of scientific formal context, followed by the beginning of a sentence (respectively, “A vaccine against coronavirus with an efficacy of 95%: …” and “The *p* value returned by a statistical test: …”) that could be completed by six possible claims simultaneously presented in a random order (for example “is 95% safe: has only a 5% risk of inducing side effects” and “established the existence of a necessarily noteworthy effect”, respectively, see Fig. 1A and B for the question format and Figs. 2 and 3 for full questions’ content). Claims about *p* value and COVID vaccine were developed according to classical definitions and misconceptions (Colquhoun, 2017) and most recent literature (Baden et al., 2021; Haute Autorité de Santé, 2020; Olliaro, 2021; Polack et al., 2020), respectively. Participants were instructed that several claims could be correct, and they had to give their answer (true/false) and their judgment of confidence on each proposed claim in order to complete the exercise. There were 12 proposed claims (6 for *p* value and 6 for vaccine efficacy).

Participants’ answers were collected through visual analog scales (VAS) ranging from “I am sure that this claim is incorrect” to “I am sure that this claim is correct” with “I do not know” in the middle of the scale (slider’s default position). VAS participant’s inputs were saved as a numerical value ranging from − 100 to + 100 (apart from the − 100, 0 and + 100 labels, no other numerical value was displayed, see illustration Fig. 1B). This double-sided VAS allowed to simultaneously collect the participants’ answer and confidence judgment (Rollwage et al., 2020).

#### Exercise 3: test results interpretation exercise

This later exercise was composed of two theoretical questions about sensitivity and specificity, one PPV calculation task and then, three last theoretical questions about PPV, NPV and prevalence (order chosen to avoid reminding the participants with these key definitions before PPV calculation task; Fig. 1A).

##### Theoretical questions

The five theoretical questions (about sensitivity, specificity, PPV, NPV and prevalence), extracted from the Quick Risk Test (Jenny et al., 2018), aimed at evaluating and controlling participants’ knowledge on necessary concepts for test results interpretation. Participants were asked to associate sensitivity, specificity, PPV and NPV concepts to their definition, among four possible and to identify prevalence as a necessary piece of information to perform PPV calculation. Questions were built according to the same structure as the two previous exercises, participants answered using the same double-sided VAS.

##### PPV calculation task

The PPV calculation task was presented in a slightly different manner than the previous exercises. One goal of this practical calculation task was to assess the impact of statistical problem framing on accuracy and confidence. Each participant was presented with a sentence of scientific formal context mentioning the sensitivity and specificity values for COVID-19 antigenic test (90 and 99%, respectively) and randomly assigned to the conditional probabilities (CP) or the natural frequencies (NF) framing group. Participants assigned to the conditional probabilities (CP) condition were told that “the test sensitivity is 90%” for instance, while participants assigned to the natural frequencies (NF) condition were told that “36 out of 40 patients who have the disease received a positive test result” (which represents the exact same information framed differently; see *Annex* in Additional file 1 for full the content of questions and interventions). When asked “If the test result is positive, what is the probability that the tested patient truly has COVID-19?” (i.e., perform a PPV calculation), participants had to provide their answer using two VAS. The first VAS controlled a slider moving from 0 to 100% with numerical feedback (answer), the second VAS allow adjusting a more (low confidence) or less (high confidence) wide blue zone of uncertainty around the first chosen value (judgment of confidence default position was set on the middle of both VAS, see Fig. 1C, see Additional file 1 for more information).

After each exercise, participants were asked to rate the subjective difficulty (from 1: very easy to 5: very difficult) and the willingness they would have had to seek for more information or to ask for help to deal with a similar situation in real-life (Yes / No) (see Fig. 1A*, “Qualitative evaluation”*).

At the end of the survey, participants were then presented with all the correct answers for the three exercises and subsequently randomly assigned to one out of three possible teaching interventions. The interventions provided explanations (text and a tree-shaped visual representation) about how to perform the PPV calculation task, framed in three alternative manners: CP framing, NF framing or the correct answer alone without further explanation. The CP- and NF-framed explanations were translated to French from previous experiments (Hoffrage & Gigerenzer, 1998; Jenny et al., 2018; Wegwarth et al., 2012). At the end of the experiment, participants were invited to subscribe to an optional second phase (by providing an email address for later contact). This second phase aimed at evaluating the impact of CP vs. NF framing of explanations during the first phase on participants’ accuracy and confidence in a similar forthcoming PPV calculation task. Participants who agreed to take part in the second phase were contacted six weeks after the first phase was completed. Phase 2 consisted in only one PPV calculation task; the instructions were presented at the same time with both framings (CP and NF) and followed by qualitative questions.

### Survey settings

The survey was conducted through the Qualtrics platform, the subscription and invitation to the second phase through a RedCap protocol in order to ensure the security of participants’ personal data. Once participants validated their response to a question, it was not possible for them to come back and change it. Among all questions and exercises, only the demographic questions and the PPV calculation task were compulsory to proceed further in the survey. All other questions were optional.

### Survey diffusion

#### Target participants and recruitment

Our target population was French-speaking medical students, residents and practicing physicians. We recruited participants from August 2nd, 2021, until February 2nd, 2022 through direct contact, professional mailing-lists and social media (Twitter, LinkedIn and Facebook mainly). Participants were volunteers; they received no financial compensation for their participation. To ensure wide distribution and foster participation, the survey was framed as a gamified quiz and we highlighted the benefits of its educational content.

#### Sample size

The information provided in the literature did not allow for a robust a priori power analysis (Cohen, 1988; Faul et al., 2007). Previous survey studies investigating statistical (il)literacy among clinicians typically included 50 to 60 participants for a between-subjects experimental design (Casscells et al., 2010; Hoffrage & Gigerenzer, 1998), and reported medium to large effect sizes on accuracy difference depending on the statistical data framing (Bramwell et al., 2006; Gigerenzer & Hoffrage, 1995). Given our design looking at several demographic variables that we suspected to impact accuracy (especially participant profiles) and associated confidence, introducing two statistical framing conditions randomized between participants for the PPV calculation task and aiming at assessing a subset of participants during a second phase, we set a high sample size target to ensure having enough power. Thus, as mentioned in the pre-registration of our study,^1^ our minimum target sample size was 300 participants. Ideally, 600 participants should complete the study in order to run Phase 2 with enough statistical power (expecting 10–15% of participants willing to do the second phase). Our maximum target sample size was 1200 participants.

### Analysis

In order to minimize data loss, we used the greatest number of participants’ responses available for each analysis. Participants that did not answer any claim of a given exercise were excluded from the analysis of this exercise.

All statistical analyses were conducted using R version 3.6.1 (R Development Core Team, 2019). Continuous variables were expressed as mean and standard deviation (sd) for normally distributed data, or as median and interquartile range (IQR) for non-normally distributed data, while categorical data were reported as frequencies and percentages. Intergroup comparisons were performed using Welch’s two-sample t-test, paired t-test, Wilcoxon–Mann–Whitney test, Chi-square test, one-way analysis of variance (ANOVA) and Kruskal–Wallis test as appropriate.

All tests were two-sided, and the level of statistical significance was set at *p* or adjusted *p* < 0.05 for all tests.

## Results

### Demographics

Overall, 898 clinicians participated in the survey (demographic questions + at least one exercise). Participants were distributed as follows: 522 medical students, 151 residents, 22 students/residents from abroad and 203 physicians. More than half of the participants were between 20 and 25 years old. The median age category was 20 to 25 [IQR = 20 to 25–30 to 35]. The proportion of women participants significantly varied across participants’ profiles, dropping from 71% among students to 66.9% among residents and finally to 42.8% among physicians (*χ*^2^ = 49.3, 2 degrees of freedom [d*f*], *p* = 1.9e−11). This variability is in line with the current women proportion among the French medical field: 70% in first year of medical studies in 2019,^2^ 61.2% among residency national exams in 2021^3^ (*χ*^2^ = 1.8, d*f* = 1, *p* = 0.18), 44.2% among practicing physicians in 2016^4^ (*χ*^2^ = 0.10, d*f* = 1, *p* = 0.74). General practice, neurology and intensive care medicine were the most represented medical specialties. Among physicians, more than one half reported working in a public hospital, and one half reported having no research activity at all, while 67% of those who reported having a research activity declared to spend less than 1 day/week on research activity (Table 1 for more details about participants’ demographics).

**Table 1:** Demographics from all participants (N = 898)

Variable	All (898)	Student (522)	Foreign student/resident (22)	Resident (151)	Physician (203]
Gender	Woman: 553 (63.3%)	Woman: 363 (70.6%)	Woman: 14 (63.6%)	Woman: 99 (66.4%)	Woman: 83 (41.9%)
	Man: 316 (35.8%)	Man: 148 (28.8%)	Man: S (36.4%)	Man: 49 (32.9%)	Man: 111 (55.1%)
	Unknown: S (0.9%)	Unknown: 3 (0.6%)	Unknown: 0(0%)	Unknown: 1 (0.7%)	Unknown: 4 (2%)
Age	Median = 20 to 25 [IQR = 20 to 25—30 to 35]	Median = 20 to 25 [IQR = 20 to 25–20 to 25]	Median = 25 to 30 [IQR = 20 to 25—25 to 30]	Median = 25 to 30 [IQR = 20 to 25 -25 to 30]	Median = 35 to 40 [IQR = 30 to 35 -45 to 50]
Experience (year)		1:15 (2.9%)		1:87 (57.6%)	Less than 5 years: 60 (30.3%)
		2: 67 (12.8%)		2: 21(13.9%)	5 to 10 years: 45 (22.7%)
		3: 74 (14.2%)		3: IS (11.9%)	10 to 15 years: 30 (15.2%)
		4:143 (27.4%)		4:16 (10.6%)	15 to 20 years: 16 (S.1%)
		5:96 (18.4%)		5:8(5.3%)	20 to 25 years: 11 (5.6%)
		6:127(24.3%)		6:1 (0.7%)	25 to 30 years: 10 (5.1%)
					30 to 35 years: 13 (6.6%)
					35 to 40 years: S (4%)
					more than 40 years: 5 (2.5%)
Specialty (top 5)		1 don't know yet: 115 (22%)		General practice: 46 (30.5%)	General practice: 48 (23.6%)
		General practice: 50 (9.6%)		Neurology: 15 (9.9%)	Neurology: 34 (15.7%)
		Surgery: 34 (6.5%)		CCM /anesthesiology: 11 (7.3%)	CCM /anesthesiology: 25 (12.3%)
		Pediatrics: 17 (3.3%)		Psychiatry: 9 (6%)	Psychiatry: 15 (7.9%)
		Anesthesia—int. care: 16 (3.1%)		Emergency medicine: 7 (4.6%)	Public health: 8 (3.9%)
Type of practice (top 3)					In a public hospital: 117 (57.6%)
					In a medical office: 39 (19.2%)
					In a private hospital: IS (S.9%)
Research time (%)					[0,20): 136 (67%)
					[20,40): 28 (13.8%)
					[40,60): 16 (7.9%)
					[60,SO):5 (2.5%)
					[SO, 100]: 13 (6.4%)
					Unknown:5 (2.5%)

Among all participants, 657 (73.2%) went through the whole survey (see Additional file 1: Fig. S2). We observed a similar attrition rate across students, residents and physicians throughout the survey, ranging from 45 to 52% (*χ*^2^ = 5.7, d*f* = 3, *p* = 0.13). Median duration completion was about 18 min and 30 s [IQR = 13′17″—27′06″]. Response profiles on test questions (see Additional file 1: Fig. S1) strongly suggest that participants have properly understood and correctly used the double-sided VAS to report both their answer and their confidence judgment (see Fig. 1, additional details in the Additional file 1).

### Exercises 1 & 2: vaccine efficacy and *p* value exercises

The results of these two exercises were analyzed by calculating, for each claim, the distance to the correct answer (*d*) defined as a score ranging from 0 to 100 containing both responses dimensions: namely the correctness of the answer (correct or incorrect) as well as the participant’s confidence in that answer obtained through the VAS. Distance to the correct answer formula was: *d* =|c*sign(*x*)*100 − *x*|/2, with *x* being the participant’s response obtained from the double-sided VAS ( − 100 < *x* < 100; *x* > 0 for claims considered as “true” and *x* < 0 for claim considered as “false”), and *c* standing for the correctness of the participant’s response (*c* = 1 for correct, *c* =  − 1 for incorrect). Consequently, while *d* = 0 stands for a correct answer with maximal confidence, *d* = 100 indicates an incorrect answer with maximal confidence. Similarly, *d* < 50 correspond to correct answers, while *d* > 50 correspond to incorrect answers. *d* = 50 stands for “I do not know” answers and, finally, *d* close to 50 corresponds to a lower level of confidence and close to 0 or 100 corresponds to a high level of confidence.

We observed a wide variability in accuracy throughout claims, ranging from 37.7 to 91% in the “vaccine efficacy” exercise, and from 28.1 to 71.4% in the “*p* value” exercise. The overall confidence in provided responses was high (median confidence per participant measured between 0 and 100: 78.5 [IQR = 65.7–90.8]) both for correct (median = 88.3 [IQR = 75.8–98.0]) and incorrect answers (median = 66.7 [IQR = 44.8–86.0]).

Three different patterns of responses emerged from Figs. 2 and 3:

- *Easy claims* resulting in a consistent correct response profile across participants (high accuracy and high confidence, overall),
- *Hard claims* resulting in a less consistent erroneous response profile (mostly low-to-high-confident incorrect answers),
- *Intermediate claims* resulting in a cleaving response profile (bimodal distribution of the accuracy with high numbers of correct and incorrect answers and a peak of low-confident uncertain responses).

More detailed discussion on these results is available in the Additional file 1.

**Fig. 4:**
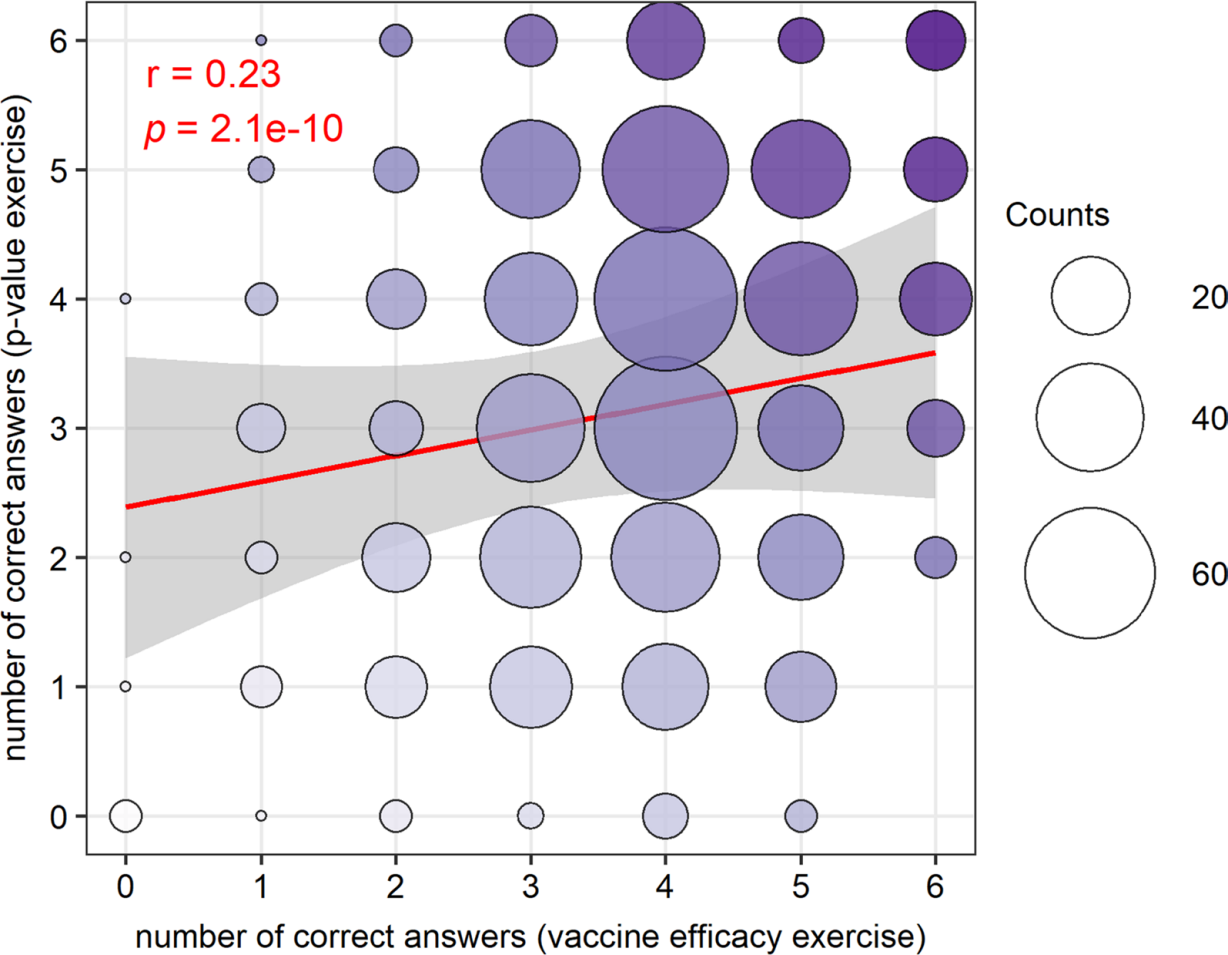
Bubble plot with regression line of the number of correct answers on the *p* value exercise as a function of number of correct answers on the vaccine efficacy exercise. There were six claims in each exercise. Performance correlated on the two exercises across participants (r and *p* indicate Pearson’s correlation coefficient and statistical significance). The red line represents the linear regression line with shaded gray area illustrating the 95% confidence interval. The circles are proportional to the number of responses in each of the cases and are for display purposes. The color intensity increases with the number of correct answers over the two exercises (from 0 in white to 12 in dark purple). All participants that completed both exercises are pooled together for this analysis (N = 756). Performance (number of correct answers out of the 6 claims) correlates between the exercises focusing on vaccine efficacy and *p* value

Among the 756 participants that completed both exercises 1 and 2, we observed a significantly positive correlation between performance (average number of correct answers across the 6 claims of each exercise) in the “vaccine efficacy” and “*p* value” exercises (Pearson’s correlation: *r* = 0.23, *p* = 2.1e−10, see Fig. 4).

Interestingly, the sub-group of 80 participants (10%) with a plausibly more advanced level in statistics (with a bachelor/master degree in mathematics, a PhD degree or practicing a research activity for at least 20% of their time) performed significantly better only at the “*p* value” exercise than the rest of respondents (lower distance to the correct answer: 31.2 for participants with advanced level in statistics, as compared to 40.1 for others; Welch’s t-test, *t* = 3.69, d*f* = 92.5, *p* = 0.0004).

#### Confidence increases more rapidly than performance

**Fig. 5:**
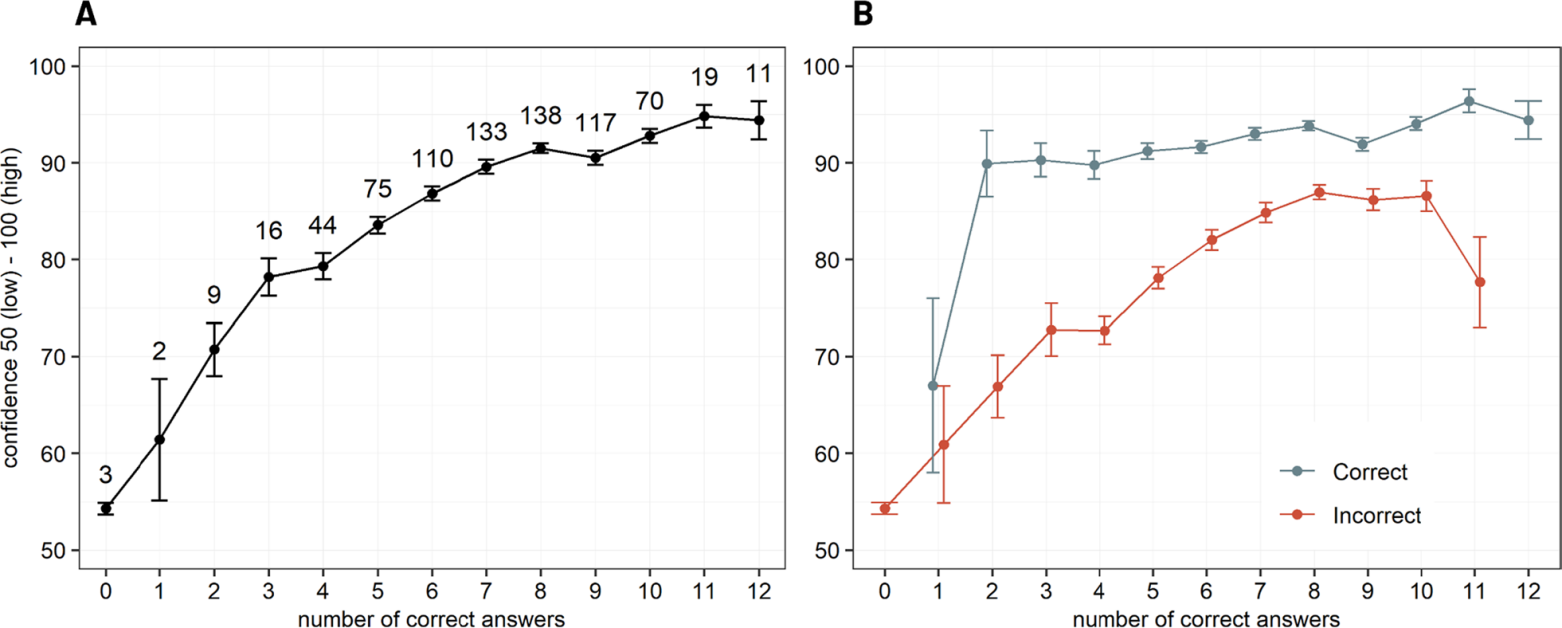
Confidence as a function of performance (number of correct answers) across the 12 claims of exercises on vaccine efficacy and *p* value (N(A) = 756, N(B) = 756). The collected data are here mapped onto a probability scale from 50 (lowest confidence, the participant answered randomly) to 100 (maximal confidence judgment). This was done by applying the transformation / 2 + 50 to the collected confidence judgments. **A** Confidence judgments of all claims from both exercises were averaged for each participant to indicate their global confidence. The linear plot shows the mean progression of these individual global confidence levels in function of the participants’ respective performance (number of correct answers) across the two exercises. Error bars indicate standard error of the mean (SEM). The values above each mean point indicate the number of participants with X correct answers. Global reported confidence increases more rapidly than performance across exercises on vaccine efficacy and *p* value. **B** Confidence judgments plotted separately for correct and incorrect answers as a function of performance (number of correct answers). Error bars indicate SEM over participants. Values for participants with 0 and 1 correct answers were, respectively, plotted according to 3 and 2 participants’ data points. Overall, confidence in correct answers was significantly higher than in incorrect answers for all levels of performance superior to one correct answer

To explore participants’ confidence as a function of performance, we analyzed participants’ mean confidence as a function of the number of correct answers over the 12 claims (ranging from 0 to 12). Figure 5A shows how mean overall confidence gradually and rapidly increases with the number of correct answers. While participants with less than two correct answers had a lower confidence score, this confidence score turned on average above 50/100 as soon as participants reached 3/12 correct answers, until reaching 80/100 for 7/12 correct answers or more. This nonlinear link between confidence and performance reveals a miscalibration in the direction of overconfidence.

#### Discrimination between correct and incorrect answers

We investigated participants’ discrimination by looking at the difference between confidence in correct vs. incorrect answers at the individual level (see Fig. 5B). Confidence was analyzed with regard to the correctness of answers (2 levels: correct/incorrect), the performance (12 levels: number of correct answers) and the participants’ profile (3 levels: students/residents/physicians) by fitting a linear mixed-effects model. The model also included gender (2 levels: men/women), practice of research (2 levels: yes/no) and the exercise topic (vaccine efficacy/*p* value) for covariates adjustment. The participant identifier was assigned as a random effect (intercept) to account for the repeated responses by participant. Significance for main effects and interactions of correctness, performance and profile was assessed based on Type II Wald Chi-square tests using the function Anova in the car R package. Post hoc pairwise comparisons were carried out on the significant higher-level interactions with the emmeans R package (https://cran.r-project.org/web/packages/emmeans/emmeans.pdf) to further determine where the differences occur across the subgroups. *P* values resulting from the post hoc tests were adjusted with the Tukey’s method to account for the multiplicity of contrasts.

This analysis revealed a three-way interaction correctness × performance × participant’s profile (Wald *χ*^2^ = 36.9, d*f* = 18, *p* = 0.005). The observed variation of discrimination across performance appears stronger in students and to a lesser extent among residents compared to physicians (see Additional file 1: Table S2C and Fig. S3). Post hoc tests showed significant differences between confidence in correct vs. incorrect answers for each level of performance (from 2 to 11 correct answers) (all adjusted *p* < 0.0001, see Additional file 1: Table S2B). However, the magnitude of the mean confidence differences between correct and incorrect answers decreased when the performance increased, dropping from 32.5 points for participants with only 3 correct answers out of 12, to 14.2 points for participants with 8 correct answers (see Fig. 5B, Additional file 1: Fig. S3 and Table S2B). This decrease of metacognitive sensitivity while performance increases relates with the gradual rise of confidence in incorrect answers with overall performance. Indeed, the confidence in correct answers quickly jumps and stays stable around 80–90/100 as soon as the performance reaches 3 out of 12 correct answers (see Fig. 5B).

In summary, the correct definitions of the concepts of vaccine efficacy and *p* value were not mastered by half of the participants who overall showed a low accuracy associated with overconfidence. However, participants’ confidence judgment allowed to discriminate between their correct and incorrect answers.

### Exercise 3: test results interpretation exercise

Six hundred eighty-one participants (63.8% of women) completed this exercise, among which 401 medical students, 108 residents, 16 students/residents from abroad and 156 physicians. The exercise participants’ demographics were similar to those of the total pool of participants (see Table 2 for more details about participants’ demographics).

**Table 2:** Demographics from participants of the “test results interpretation” exercise (N = 681)

Variable	All (681)	Student (401)	Foreign student/resident (16)	Resident (108)	Physician (156)
Gender	Woman: 432 (53. 8%)	Woman: 284 (71.2%)	Woman: 9 (56.2%)	Woman: 73 (67.6%)	Woman: 66 (42.9%)
	Man: 239 (35.3%)	Man: 113 (28.3%)	Man: 7 (43.8%)	Man: 34 (31.5%)	Man: 85 (55.2%)
	Unknown: 6 (0.9%)	Unknown: 2 (0.5%)	Unknown: 0 (0%)	Unknown: 1 (0.9%)	Unknown: 3 (1.9%)
Age	Median = 20 to 25 [IQR = 20 to 25–30 to 35]	Median = 20 to 25 [IQR = 20 to 25–20 to 25]	Median = 25 to 30 [IQR = 20 to 25–25 to 30]	Median = 25 to 30 [IQR = 20 to 25–25 to 30]	Median = 35 to 40 [IQR = 30 to 35–45 to 50]
Experience (year)		1:10 (2.5%)		1: 6c (61.1%)	Less than 5 years: 47 (30.5%)
		2: 47 (11.7%)		2: 11 (10.2%)	5 to 10 years: 37 (24%)
		3:5S (14.5%)		3:15 (13.9%)	10 to 15 years: 22 (14.3%)
		4:107 (26.7%)		4:11 (10.2%)	15 to 20 years: 12 (7.8%)
		5:75 (1S.7%)		5: 5 (4.6%)	20 to 25 years: 9 (5.8%)
		6:104 (25.9%)			25 to 30 years: 6 (3.9%)
					30 to 35 years: 10 (6.5%)
					35 to 40 years: 7 (4.5%)
					More than 40 years: 4 (2.6%)
Specialty (top 5}		1 don't know yet: 85 (21.2%)		General practice: 36 (33.3%)	Neurology: 31 (19.9%)
		General practice: 39 (9.7%)		Neurology: 13 (1Z%)	General practice: 30 (19.2%)
		Surgery: 23 (5.7%)		CCM/anesthesiology: 9 (8.3%)	CCM/anesthesiology: 20 (12.8%)
		Pediatrics: 15 (3.7%)		Surgery: 5 (4.6%)	Psychiatry: 13 (8.3%)
		Anesthesia—int. care: 12 (3%)		Pediatrics: 4 (3.7%)	Public health: 6 (3.8%)
Type of practice (top 3)					In a public hospital: 94 (60.3%)
					In a medical office: 26 (16.7%)
					In a private hospital: 15 (9.6%)
Research time (%)					[0,20): 104 (66.7%)
					[20,40): 21 (13.5%)
					[40,60): 14 (9%)
					[60,80): 4 (2.6%)
					[80,100]: 11 (7.1%)
					Unknown: 2(1.3%)

#### Theoretical questions

In this exercise, participants were asked to identify the correct definitions of sensitivity, specificity, PPV and NPV. For each question, they had to evaluate four different options (see Additional file 1: Fig. S4). Responses to these (non-mandatory) questions reveal that 80.4% (512/637) and 66.8% (429/642) of participants identified the correct definitions of sensitivity and specificity, respectively, while 75.7% (442/584) and 73.9% (437/591) correctly identified PPV and NPV. Only 68.4% (372/544) of the participants knew that prevalence is required to perform PPV calculation. In all five questions, options were correctly selected or rejected with almost maximum confidence (median = 100 [IQR = 100–100]), while confidence in incorrect answers was more variable (see Additional file 1: Fig. S4). If 6.2% of participants misattributed specificity definition to sensitivity, 15.9% did the opposite (attributing sensitivity definition to specificity).

#### PPV calculation task

PPV calculation aimed at measuring participant’s accuracy and confidence on a related practical task. The correct PPV was 26%. We considered all answers included in the 26 ± 5% range as correct. The vast majority of participants performed this difficult task (only 3% of participants barely moved the VAS cursors within the 50% ± 5% zones and thus, were kept in the analysis). The PPV calculation task was analyzed according to correctness, confusion with sensitivity/specificity, and instruction framing (NF/CP) using Wilcoxon–Mann–Whitney test, Kruskal–Wallis test and Chi-square test or two-way ANOVA where appropriate.

#### General accuracy and confidence

**Fig. 6:**
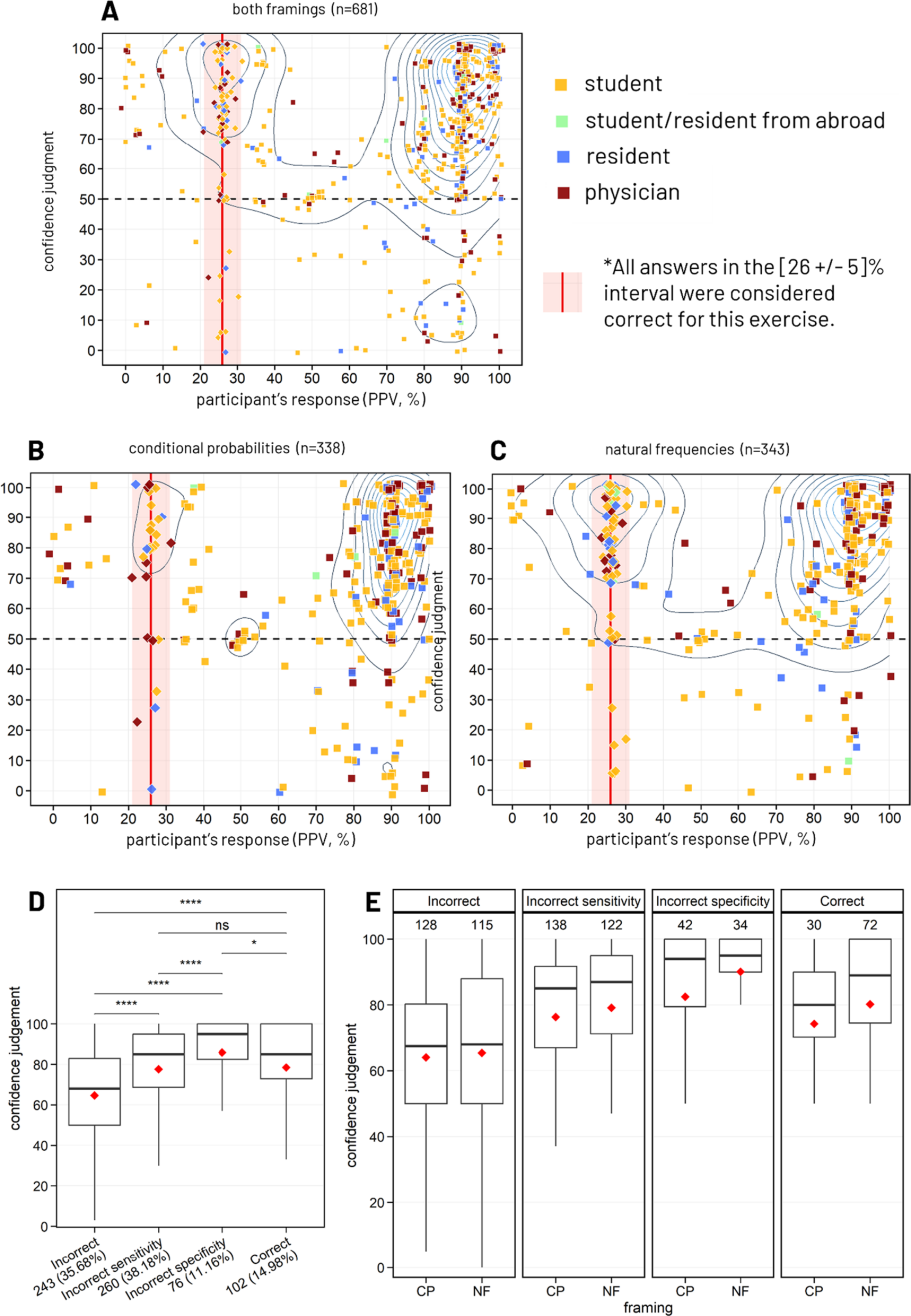
Responses to predictive positive value calculation task, N = 681. Distribution of participants’ predictive positive value (PPV) estimation and their associated confidence judgment ranging from 0 (low confidence) to 100 (high confidence). Overall accuracy was 15% (**A**) and dependent on the problem framing: 9% in conditional probabilities (**B**) & 21% for natural frequencies (**C**). The red line stands for the PPV (26%); all answers included in the 26 ± 5% interval were considered correct. Density contour lines highlight the most represented answers and confidence judgments among participants. In the task instructions, sensitivity and specificity were, respectively, 90 and 99%. **D** Boxplots comparing the confidence judgments by response correctness. Means are indicated by a red diamond shape and the counts and percentages are given in the group labels. Post hoc comparisons using Tukey’s method (emmeans R package). ****p value < 0.0001, *p value < 0.05, NS: non-significant (*p* value > 0.05). (E) Boxplots representing the confidence judgments of respondents by framing and response correctness. Means are indicated by a red diamond shape and counts are given on top of the plots. Tests based on a multivariate linear regression model indicated an effect of the factors “correctness” and "framing" with no interaction effect between the two factors. Type II ANOVA (F-tests) (car R package)

Only 15% (102/681) of the participants solved this task correctly (see Fig. 6A). The overall confidence in responses (from 0 to 100) was high (median = 80 [IQR = 61–95]). Interestingly, Fig. 6A reveals 2 clusters of incorrect answers around 90% and 99%. These incorrect answers might be explained by confusion with sensitivity or specificity, respectively. For further analysis, we isolated “confusion with sensitivity” responses defined as answers within the 90 ± 1% range, and “confusion with specificity” defined as answers within the 99 ± 1% range. More than a third (38.2%) of all answers (260/681) fell in the zone of confusion with sensitivity, representing 45% of all incorrect answers (260/579). Similarly, 11.2% of all answers (76/681) fell within the zone of confusion with specificity, representing 13.1% of all incorrect answers (76/579). Among the 49 participants who misattributed sensitivity definition to PPV in the theoretical questions, 57.1% (28/49) also confused the PPV for the sensitivity in the calculation task, which is significantly more than incorrect answers attributable to confusion with sensitivity among the participants who knew the PPV definition (33.5%, 148/442, *χ*^2^ = 9.7, d*f* = 1, *p* = 0.002).

The participants who found a correct answer in the PPV task performed slightly better on the 2 previous exercises about “vaccine efficacy” and “*p* value” (by having more correct answers across the 12 claims (median = 8 [IQR = 7–10] vs. participants that did not correctly solve the PPV calculation: median = 7 [IQR = 6–9], Wilcoxon–Mann–Whitney test, *p* = 0.0007). Of note, the sub-group of 73 participants (10%) with a plausibly more advanced level in statistics did not perform better than the rest of respondents on this practical task.

#### Accuracy according to framing

Participants were twice more likely to give a correct answer when the problem was framed in natural frequencies when compared to conditional probabilities (NF: 21%, 72/343 vs. CP: 8.0%, 30/338; *χ*^2^ = 18.7, d*f* = 1, *p* = 1.5e−5, see Figs. 6B and C). A major distinction between the two framings lies in the fact that sensitivity and specificity values are explicitly displayed in conditional probabilities instructions, whereas they only can be inferred by the numerical pieces of information provided in natural frequencies condition. Despite this discrepancy, we found no difference in confusion rate with sensitivity (NF: 122/343, 35.6% | CP: 138/338, 40.83%, *χ*^2^ = 1.8, d*f* = 1, *p* = 0.18) nor with specificity (NF: 34/343, 9.9% | CP: 42/338, 12.4%, *χ*^2^ = 0.8, d*f* = 1, *p* = 0.36) depending on the framing, suggesting that confusion between PPV and sensitivity or specificity is not mitigated by how the information is framed.

#### Discrimination by confidence at the group level

Group differences in confidence judgment regarding the type of correctness (4 levels: correct, incorrect, incorrect attributable to a confusion with sensitivity, incorrect attributable to a confusion with specificity), the framing (NF vs. CP) and their interaction term were assessed using a multiple linear regression model. Significance of both factors was tested using a Type II ANOVA (F-tests) from the car R package, and post hoc comparisons were performed with the Tukey’s method in the emmeans R package. Prior to modeling, the values of confidence were transformed by the function y = sqrt(100 − x) to accommodate the assumptions of normality and variance equality of the residuals. F-tests indicated a main effect of correctness (F[d*f* = 3, 673] = 29.5, *p* < 0.0001, see Fig. 6D and Additional file 1: Table S3A) and of the framing (F[1, 673] = 4.0, *p* = 0.045) with no interaction (F[3, 673] = 0.6, *p* = 0.58, see Fig. 6E and Additional file 1: Table S3B). Based on estimated marginal means (i.e., group means estimated by the model) with standard error (SE), confidence judgments for answers given with the NF framing were slightly greater than with the CP framing (85.1 ± 1.0 vs. 82.0 ± 1.2, respectively). The confidence in incorrect responses attributable to confusions (with sensitivity: 83.9 ± 1.3, or with specificity: 93.4 ± 1.5) was found to be higher than confidence in other incorrect responses (70.7 ± 1.8; both adjusted *p* < 0.0001), and rather similar (or higher) to confidence in correct answers (86.3 ± 1.9) (see Fig. 6D and Additional file 1: Table S3C).

#### Perceived difficulty and propensity to ask for help

A higher perceived level of difficulty of the PPV calculation task was significantly associated with an increased clinician’s propensity to be willing to look for help if they were facing this task in a real-life situation (χ^2^ = 64.1, d*f* = 4, p = 4.0e−13). Participants were also more prone to be willing to look for help when giving an incorrect answer not attributable to a confusion with sensitivity or specificity (84.0%, 199/237) when compared to those that gave a correct answer (71.6%, 73/102) or an incorrect answer attributable to a confusion with sensitivity or specificity (74.6%, 244/327, *χ*^2^ = 9.3, d*f* = 2, *p* = 0.01).

#### Study follow-up

As expected, only a small proportion of the participants (9.5%) that fully completed the main study signed up for the second phase. The group constituted of the 65 participants that filled both phases was better at solving the phase 1 PPV calculation task (28/65, 43.1% vs. 102/681, 15.0%, hypergeometric test for over-representation, *p* = 5.8e − 9). The global accuracy did not significantly increase between phase 1 (28/65) and phase 2 (32/65, *χ*^2^ = 0.3, d*f* = 1, *p* = 0.60). Unfortunately, the small sample size did not allow to show any effect of the teaching material randomly assigned at the end of the phase 1.

In summary, despite a high accuracy in theoretical questions (definitions related to test results interpretation), we observed an overall low accuracy in the practical PPV calculation task. Most incorrect answers were attributable to a confusion between PPV and sensitivity or specificity. Moreover, these confusions were associated with the same level of confidence as for correct answers. NF framing was associated with about twice the proportion of correct answers when compared to CP framing with only a marginal effect on overconfidence (irrespective of answer correctness).

## Discussion

### Interpretation

#### Statistical illiteracy among clinicians

In this work, we studied statistical literacy among clinicians with a focus on their confidence and its discrepancy with their accuracy. In line with previous work reporting statistical illiteracy among clinicians (Anderson et al., 2014; Bramwell et al., 2006; Gaissmaier & Gigerenzer, 2008; Hoffrage & Gigerenzer, 1998; Jenny et al., 2018; Labarge et al., 2003; Wegwarth, 2013; Wegwarth et al., 2012), we also observed a low to medium accuracy on numerous theoretical claims and practical tasks related to statistics commonly encountered in the medical context. Surprisingly, despite the omnipresence of the question of COVID-19 vaccines’ efficacy among the general public, the caregivers and in the media at the time of the study (08/2021–02/2022) and the widespread question of *p* values interpretation in medical articles, only a half of participants could identify their correct definition. Our results further show that although clinicians strongly master theoretical knowledge required to perform test results interpretation, it did not prevent them from failing the PPV calculation task with a high confidence. The rate of incorrect answers for the PPV calculation was similar to those of previous studies and also mostly related to a systematic confusion of PPV with sensitivity (or, less frequently, with specificity) (Gigerenzer et al., 2007; Hoffrage & Gigerenzer, 1998). Our results show that these biased responses toward sensitivity or specificity led to confidence levels as high as for correct answers. The tendency to confuse PPV with sensitivity rather than specificity might be due to the relative similarity between PPV’s and sensitivity’s definitions (both entailing “positive test results” and “sick patient”). Such a low proficiency in statistics could directly impact clinical decisions.

#### Misalignment of confidence and accuracy

The observed overall low accuracy was associated with a high confidence. Participants were confident in both their correct and incorrect answers. Although previous experimental paradigms have been criticized, many studies already highlighted clinicians’ miscalibration toward overconfidence (Ahmed & Walsh, 2020; Barnsley et al., 2004; Berner & Graber, 2008; Borracci & Arribalzaga, 2018; Brezis et al., 2019; Davis et al., 2006; C. Friedman et al., 2001; C. P. Friedman et al., 2005; Lam & Edward Feller, 2020; Lawton et al., 2019; Meyer et al., 2013; Miller et al., 2019; Morgan & Cleave-Hogg, 2002; Naguib et al., 2019; O’Donoghue et al., 2018; Rahmani, 2020). However, to our knowledge, our study is the first to explore such misalignment between confidence and general accuracy in knowledge about statistics while evaluating at the same time clinicians’ discrimination abilities through their confidence judgments. Overall, the overconfidence bias observed in exercises about vaccine efficacy and *p* value was mainly driven by increased confidence in incorrect answers among participants with intermediate performance. Our results also confirm the preliminary insights of high confidence in incorrect PPV estimations shown in a previous study (Bramwell et al., 2006). These results challenge the qualitative feedback collected among participants of the original study from Hoffrage and Gigerenzer, (1998) that some physicians were aware of their statistical illiteracy (they reported to feel “embarrassed by their innumeracy and trying to hide it from their patients”) (Gigerenzer et al., 2007). However, in our study, we highlight that most of the incorrect answers are highly plausibly triggered by a confusion between PPV and sensitivity or specificity: these two different kinds of errors would explain this apparent discrepancy. Our results further show that errors attributable to a confusion were associated with a higher confidence that other incorrect answers, this confidence reaching or even exceeding the confidence reported for correct answers. Altogether, these insights suggest that the phenomenon of illusion of knowledge might be at play.

#### Evidence for clinicians’ metacognitive sensitivity

Besides the misalignment between global confidence and general accuracy, we provide the first clear evidence of a substantial clinicians’ metacognitive sensitivity (measured via discrimination). Regardless of their tendency to be miscalibrated toward overconfidence, participants’ confidence in correct answers was significantly higher than in incorrect answers, irrespective of their performance. Overall, clinicians’ confidence discriminated between their correct and their incorrect answers at the individual (exercises 1 and 2) and group level (PPV calculation task in exercise 3, put aside specific errors attributable to confusions), thus showing a degree of metacognitive sensitivity. In exercises about vaccine efficacy and *p* value, discrimination dropped when performance improved. In other words, relatively bad performers (with less than 4 correctly answered claims out of 12) were more sensitive (reported a lower confidence in their numerous incorrect answers compared to their few correct answers) than relatively good performers, who were very highly confident both in their correct and incorrect answers. This difference in discrimination stems in the contrast between the constant high confidence in correct answers and the rising confidence in incorrect answers while performance increases. This variation of confidence (second-order judgments) when first-order performance fluctuates is aligned with previous observations in the experimental psychology literature (Fleming et al., 2012; Rouault et al., 2018). These new findings suggest that more research should focus on disentangling clinicians’ bias from discrimination.

#### Impact of statistical problem framing

A commonly evoked intervention to decrease the number of errors toward statistical tasks is to express the information using more intuitive framing such as natural frequencies (NF) (Gigerenzer et al., 2007). Although with a smaller magnitude than previously reported (Gigerenzer et al., 2007; Hoffrage & Gigerenzer, 1998), the NF-framed teaching strategy allowed to double the accuracy when compared to CP framing (Bramwell et al., 2006; Gigerenzer et al., 2007; Hoffrage & Gigerenzer, 1998). This framing also triggered a small although significant increase in confidence, raising a mild risk (very small effect size) of backfire of such type of intervention by strengthening the illusion of knowledge. One reason that could explain this side effect of NF framing on confidence lies in the fact that instructions look shorter in length and appear easier than CP in terms of wording and provided statistical information.

### Limitations

The present study has some limitations. First, since we did not require for official registration to medical school or institution to participate to the study, we did not formally control whether participants were actually studying or practicing medicine. However, the absence of financial compensation mitigates the risk of illegitimate participation. In addition, since our sample was composed of volunteers invited through social media, it might not be representative of the variety of the whole clinicians’ French population. Finally, the smaller size of the positive effect that NF framing had accuracy in the PPV calculation task, when compared to the seminal work ((Gigerenzer et al., 2007; Hoffrage & Gigerenzer, 1998) that enrolled a given subspecialty), could be explained by the heterogeneity of our sample.

Second, although allowing for more granularity than Likert scales, the double-sided and paired accuracy-confidence VAS are not yet commonly used in the field of medical decision-making. Nevertheless, they are more and more often used in experimental psychology and could better tackle the weighted decision process of the participants (Fleming & Lau, 2014; Galvin et al., 2003; Maniscalco & Lau, 2012; Masson & Rotello, 2009). Although allowing more granularity than Likert scales, the double-sided scales and the paired accuracy-confidence visual analog scales may have impacted reported confidence (Juslin et al., 2003). Whereas the literature is not unanimous on this topic (Dinsmore & Parkinson, 2013), we cannot completely rule out the risk of priming a certain level of confidence through the formulation of our instructions, questions and labels (specifically with the use of the words “sure” and “confident”, the expression “I do not know” and the corresponding figures). An uncertainty also lies in how to interpret the responses in the center of the double-sided VAS. Indeed, this way of measuring responses does not allow to clearly disentangle a correct “I do not know” response from responses given quickly without properly reading the instructions. Another concerning aspect of explicitly collecting confidence judgments is to in turn affect accuracy by triggering participants' metacognition. This reactivity effect could lead either to an increased accuracy (more attention paid to the task) or decreased accuracy (cognitive resources devoted to confidence judgment instead of the first-order task) (Double & Birney, 2019; Double et al., 2018; Mitchum et al., 2016). What is more, confidence judgments are also dependent on first-order performance and thus linked to individual knowledge and accuracy (Fleming & Lau, 2014; Galvin et al., 2003; Maniscalco & Lau, 2012; Masson & Rotello, 2009).

Third, by necessity when designing an experiment, several choices were made in the survey content and its analysis. In exercises on vaccine efficacy and *p* value, we originally considered each claim comparable, whereas they might not be equally difficult in reality, as the variability of accuracy across claims suggests. We did not discriminate between being (in)correct by selecting a true (false) claim from being (in)correct by rejecting a false (true) claim, whereas these might not be symmetrical situations. Although recent evidence advocates for the existence of a shared brain system, similarly computing confidence across cognitive domains (Rouault et al., 2022), our survey focused on three precise topics, which may limit the extent to which our results would generalize.

Finally, our experimental design did not rely on a high number of trial repetitions. Neither did it enable participants to practice or to get feedback in order to improve their performance and/or the relevance of their confidence judgments (Fleming & Lau, 2014; Lejarraga & Hertwig, 2021). Stimuli content and difficulty may not have been perfectly controlled nor representative of real-life clinical situations, falling in the item selection trap by creating a context prone to overconfidence observation (Gigerenzer et al. 1991). The constant high level of confidence in correct answers might be related to these differences in difficulty across claims; several very easy claims were successfully answered by most participants. Investigating confidence and its alignment with accuracy through several single-event confidence judgments might lead to the conclusion of an overconfidence bias, while collecting participants’ estimation of their frequency of correct responses would not have (Gigerenzer, 1991). While this criticism applies to studies based on diagnosis exercises using case-vignettes, we chose statistical notions that we consider directly impacting clinical decisions’ quality. We also presented them ecologically, as they are taught and framed in real life. Overconfidence is sometimes suspected to only arise from regression to the mean (Olsson, 2014).

### Highlights and perspectives

Clinicians make high-stake decisions on a daily basis, often under high levels of uncertainty, emergency, stress, fatigue and emotional or cognitive load. In addition to these contextual factors that can undermine the quality of clinical choices, medical students and clinicians’ poor proficiency toward numerical and statistical data can compromise patient safety. Our results provide new evidence of statistical illiteracy among medical staff, with a low response accuracy across three basic statistical knowledge in medicine. Our findings further indicate that these mistakes are often made with high confidence, suggesting that the phenomenon of illusion of knowledge might be at play. More specifically, while the theory is far from being mastered regarding vaccine efficacy and *p* value, most of the participants successfully answered theoretical questions about test results interpretation notions, but did not manage to correctly solve the PPV calculation task. Interestingly, beyond overconfidence bias, clinicians also showed a degree of metacognitive sensitivity, with their confidence discriminating between their correct and incorrect answers. This new finding questions the relevance of previous studies’ perspective and conclusions on the matter, and advocates for more research developing robust ecological paradigms to better disentangle clinicians’ bias from discrimination. Different corrective strategies might be considered depending on whether staff members are aware of their deficiencies (providing pedagogical content might be enough) or not (if practitioners believe they are right, the first step might be to attract their attention to the fact that they are mistaken). This is why evaluating clinicians’ judgments of confidence in their knowledge is a crucial step toward the elaboration of effective pedagogical interventions. A commonly evoked solution to address statistical illiteracy is to use more intuitive framings to present data, in particular natural frequencies. The results of our study suggest that, along with an important improvement of accuracy, the use of NF framing also induces a side effect by increasing reported confidence, both in correct and incorrect answers. We consider that the benefit of NF, which allowed to double accuracy, compensates for the minor effect size of increased confidence, and we therefore advocate that the tradeoff balances in favor of NF framing.

## Supplementary Information

**Additional file 1.** Supplementary Figures and Tables.

## Data Availability

The complete survey script (in French) and the analyzed dataset will be available upon reasonable request.

## Abbreviations

PPV: Positive predictive value
NPV: Negative predictive value
CP: Conditional probabilities
NF: Natural frequencies
VAS: Visual analog scale

## Footnotes

1 Pre-registration on OpenScienceFramework: https://osf.io/5hmcs.

2 Peltier C. (2019, February 19). En médecine, plus de filles mais toujours autant d’enfants de cadres. *Le Monde*. https://www.lemonde.fr/campus/article/2019/02/09/en-medecine-plus-de-filles-mais-toujours-autant-d-enfants-de-cadres_5421479_4401467.html

3 https://www.legifrance.gouv.fr/jorf/id/JORFTEXT000044229585

4 La démographie des professionnels de santé. DREES. https://drees.solidarites-sante.gouv.fr/sources-outils-et-enquetes/la-demographie-des-professionnels-de-sante
