# Supplementary material for: Illusion of knowledge in statistics among clinicians: evaluating the alignment between objective accuracy and subjective confidence, an online survey
Source: Cogn Res Princ Implic. 2023 Apr 20;8:23. doi: 10.1186/s41235-023-00474-1 (PMC10118231; doi:10.1186/s41235-023-00474-1)
Supplement: Supplementary file 1 — Additional file 1. Supplementary Figures and Tables. [file 41235_2023_474_MOESM1_ESM.pdf]

SUPPLEMENTARY MATERIAL

Illusion of knowledge in statistics among clinicians: evaluating the alignment between objective accuracy and subjective confidence: an online survey

Lakhlifi Camille[1,2*], Lejeune François-Xavier[1,3+], Rouault Marion[1,4+], Khamassi Mehdi[5], Rohaut Benjamin[1,6*]

1. Sorbonne Université, Institut du Cerveau - Paris Brain Institute - ICM, Inserm, CNRS, APHP, Hôpital de la Pitié Salpêtrière, Paris, France.
2. Université Paris Cité, Paris, France.
3. Paris Brain Institute's Data Analysis Core, Hôpital de la Pitié Salpêtrière, Paris, France.
4. Département d'Études Cognitives, École Normale Supérieure, Université Paris Sciences & Lettres (PSL University), Paris, France.
5. Institute of Intelligent Systems and Robotics, CNRS, Sorbonne Université, CNRS, Paris, France.
6. AP-HP, Hôpital de la Pitié Salpêtrière, DMU Neurosciences, Paris, France.

+ These authors have contributed equally.

*** Corresponding authors**:

camille.lakhlifi@icm-institute.org & benjamin.rohaut@sorbonne-universite.fr

SUPPLEMENTARY FIGURES AND RESULTS

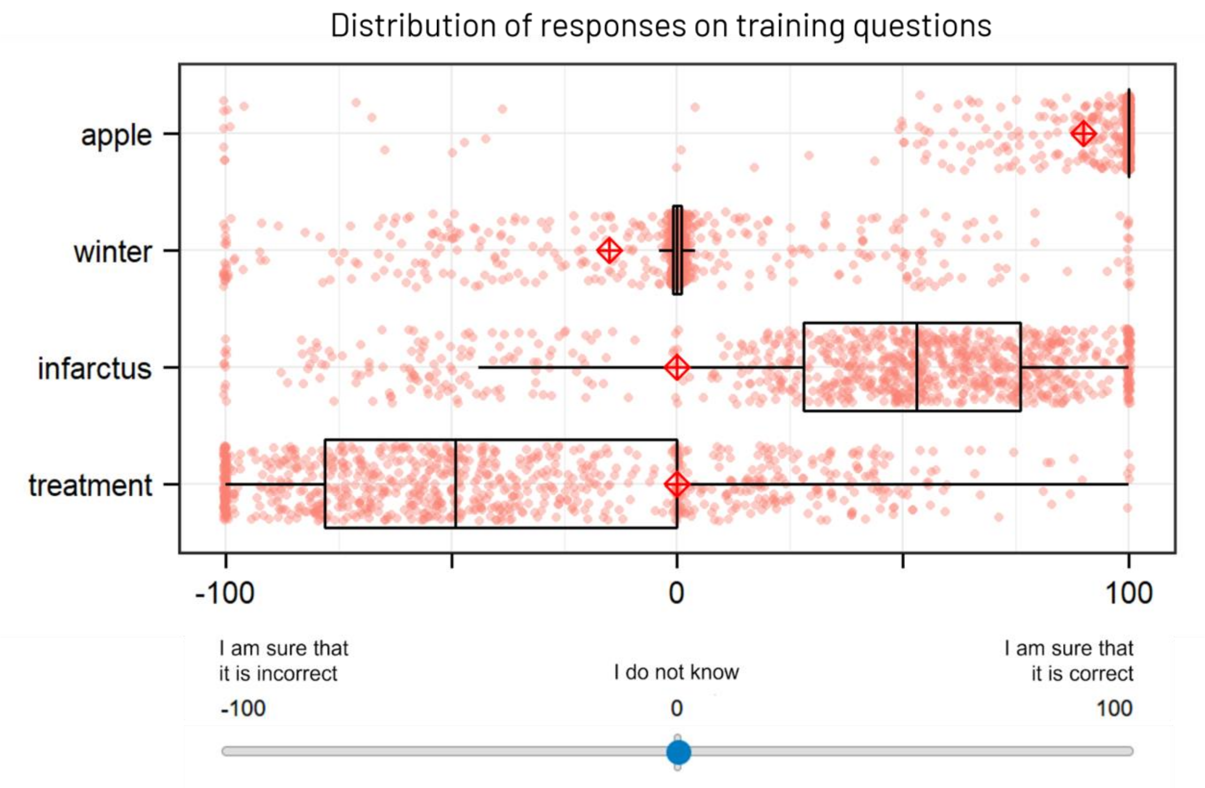

<u>Supplementary Figure 1</u>. **Validation of the measurement scale**

Before completing the survey, participants were presented with four training questions (two general and two medical, one of each supposed to trigger a confident answer and the other an uncertain answer) to get familiar with the granularity of the measurement scale:

- apple: "In general, apples can be red, yellow and/or green:"
- winter: "Winter 2021 will be colder than winter 2020:"
- infarctus: "A smoking patient in his fifties calls his physician, panicked: he just ran after his bus and suddenly feels a violent chest pain. The physician immediately thinks about myocardial infarction. What do you think about this diagnosis?"
- treatment: "One of your colleagues reads a study suggesting that a new treatment shows promising results to treat a disease X in a test population composed of patients aged between 18 and 30. The next day, he welcomes a 56 years-old patient with the disease X: your colleague thinks that there is a high chance for this treatment to work on this patient. What do you think about this reasoning?"

  Results are presented with boxplots (median and interquartile range), the red marker represents the default position of the cursor.

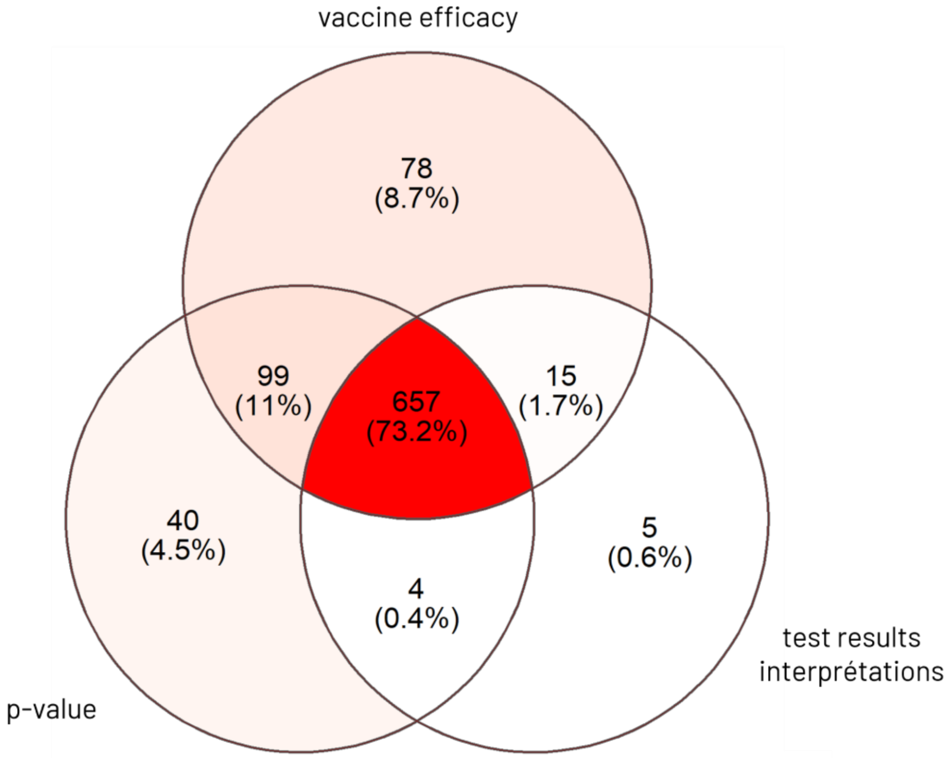

Supplementary Figure 2. Respondents' completion of the survey

Overall, 898 clinicians completed at least one exercise of the survey. 73.2% of them fully filled all exercises, with an equivalent attrition rate between physicians, residents and students. The proportion of participants that stopped the survey before completing the second exercise was greater for those who started with the "vaccine efficacy" exercise and then faced the "p-value" exercise than for the opposite order (8.7% vs 4.5% respectively, Chi-square test: $\chi2$ = 12.4, df = 1, p = 4.2e-4, see *Figure 3*).

“vaccine efficacy” exercise			
	French	*English*	**Explanation**
A	**Un vaccin contre le coronavirus dont l'efficacité est de 95% :**	**A vaccine against coronavirus with an efficacy of 95%:**	
1	- empêche de développer une forme grave de la maladie dans 95% des cas	- prevents the development of a severe form of the disease in 95% of cases	Wrong outcome: As indicated in the question, the primary outcome of vaccine efficacy studies was confirmed symptomatic disease (Haute Autorité de Santé, 2020 & 2021Baden et al., 2021; Falsey et al., 2021; Polack et al., 2020). Furthermore, the effect size of severe forms protection was unknown at that time (number of severe cases were too small to accurately estimate efficacy for severe cases.<br><br>Wrong interpretation: Efficacy is a measure of the relative reduction in risk and not an absolute measure of protection (e.g. a vaccinated person over-exposed would have a higher risk than an underexposed one, irrespectively to vaccine efficacy).
2	- empêche 95% des personnes vaccinées exposées au virus de développer la maladie	- prevents 95% of vaccinated individuals exposed to the virus from developing the disease	Right outcome.<br><br>Wrong interpretation: Efficacy is a measure of the relative risk reduction and not an absolute measure of protection. This confuses efficacy with protection to a population that can be influenced by factors such as age, immune status, strain of the virus, etc.. (Olliaro, 2021).
3 ✅	- réduit de 95% le taux d'incidence de la maladie parmi les personnes vaccinées	- reduces the incidence rate of the disease by 95% among vaccinated individuals	This is a correct answer.
4	- empêche une même personne vaccinée de développer la maladie pour 95% de ses expositions au virus	- prevents a single vaccinated individual from developing the disease for 95% of his exposures to the virus	Right outcome.<br><br>Wrong interpretation: This confuses efficacy (incidence reduction applied to a population) with individual protection that can be influenced by factors such as age, immune status, strain of the virus, etc..
5 ✅	- a été évalué dans un essai clinique afin de collecter les données nécessaires pour en calculer l'efficacité	- has been evaluated in a clinical trial to collect the data necessary to calculate its efficacy	This is a correct answer.
6	- est sûr à 95% : présente seulement 5% de risques d'induire des	- is 95% safe: has only a 5% risk of	Wrong outcome: This confuses efficacy and safety.

	effets secondaires	inducing side effects	

“p-value” exercise			
	French	*English*	**Explanation**
B	**Le "petit p" (la p-value) indiqué par un test statistique :**	**The p-value returned by a statistical test:**	
1	- permet d'affirmer que l'effet observé existe réellement si sa valeur est inférieure au seuil de comparaison alpha (fixé arbitrairement, le plus souvent à 5%)	- allows to conclude that the observed effect really exists if its value is lower than the alpha comparison threshold (fixed arbitrarily, most often at 5%)	Wrong interpretation: Inversion of conditional probability and tested hypothesis. This confounds the probability of the alternative hypothesis H1being true according to data (p(H1/data)) with the probability to observe the data (or more extreme data) under the null hypothesis (p(data/H0).
2 ✅	- représente la probabilité d'observer l'effet décelé ou un effet plus extrême par hasard dans l'hypothèse où l'hypothèse nulle (H0) est vraie	- represents the probability of observing a similar or greater effect if the null hypothesis (H0) were true	This is a correct answer (Altman & Krzywinski, 2017).
3	- permet d'établir l'existence d'un effet nécessairement digne d'intérêt	- establishes the existence of a necessarily noteworthy effect	Wrong answer: This confounds p-value with practical significance or clinical impact.
4	- représente l'importance de la différence observée entre les groupes	- represents the importance of the observed difference between the groups	Wrong answer: This confounds p-value with the size of the observed difference between the groups (i.e. effect size that can be assessed with dedicated metrics such as Cohen's d)
5	- représente la probabilité que l'hypothèse testée (H1) soit vraie	- represents the probability that the tested hypothesis (H1) is true	Wrong answer: This confounds the probability of correctly rejecting the null hypothesis H0 when it is false (i.e. power = 1-β, where β is the probability of failing to reject the H0 when it is false) with the probability to observe that data under the null hypothesis (p(data/H0).
6 ✅	- est dépendant de la taille d'échantillon testée	- is dependent on the tested sample size	This is a correct answer.

Supplementary Table 1. Explanations for right and wrong claims from exercises about “vaccine efficacy” and “p-value”.

Altman, N., & Krzywinski, M. (2017). Points of Significance: Interpreting P values. *Nature Methods*, *14*(3), 213–215.

Baden, L. R., El Sahly, H. M., Essink, B., Kotloff, K., Frey, S., Novak, R., Diemert, D., Spector, S. A., Rouphael, N., Creech, C. B., McGettigan, J., Khetan, S., Segall, N., Solis, J., Brosz, A., Fierro, C., Schwartz, H., Neuzil, K., Corey, L., … COVE Study Group. (2021). Efficacy and Safety of the mRNA-1273 SARS-CoV-2 Vaccine. *The New England Journal of Medicine*, *384*(5), 403–416. https://doi.org/10.1056/NEJMoa2035389

Falsey, A. R., Sobieszczyk, M. E., Hirsch, I., Sproule, S., Robb, M. L., Corey, L., Neuzil, K. M., Hahn, W., Hunt, J., Mulligan, M. J., McEvoy, C., DeJesus, E., Hassman, M., Little, S. J., Pahud, B. A., Durbin, A., Pickrell, P., Daar, E. S., Bush, L., … Gonzalez-Lopez, A. (2021). Phase 3 Safety and Efficacy of AZD1222 (ChAdOx1 nCoV-19) Covid-19 Vaccine. *New England Journal of Medicine*, *385*(25), 2348–2360. https://doi.org/10.1056/NEJMoa2105290

Haute Autorité de Santé. (2020). *Revue rapide sur les tests de détection antigénique du virus SARS-CoV-2*. Saint-Denis La Plaine HAS.

Olliaro, P. (2021). What does 95% COVID-19 vaccine efficacy really mean? *The Lancet Infectious Diseases*, *21*(6), 769. https://doi.org/10.1016/S1473-3099(21)00075-X

Polack, F. P., Thomas, S. J., Kitchin, N., Absalon, J., Gurtman, A., Lockhart, S., Perez, J. L., Pérez Marc, G., Moreira, E. D., Zerbini, C., Bailey, R., Swanson, K. A., Roychoudhury, S., Koury, K., Li, P., Kalina, W. V., Cooper, D., Frenck, R. W., Hammitt, L. L., … C4591001 Clinical Trial Group. (2020). Safety and Efficacy of the BNT162b2 mRNA Covid-19 Vaccine. *The New England Journal of Medicine*, *383*(27), 2603–2615. https://doi.org/10.1056/NEJMoa2034577

Specific insights by exercise

Accuracy and confidence regarding "vaccine efficacy" varied across claims. A vast majority of respondents identified with high confidence that a vaccine efficacy of 95% did not mean that the vaccine is 95% safe (91% of correct answers), and did not mean that it prevents a single vaccinated individual from developing the disease for 95% of his exposure to the virus (77.4% of correct answers). Similarly, 87.3% of the participants correctly identified that the vaccine efficacy is evaluated in a clinical trial. Cleaving response profiles arose from the three last claims. As such, participants overall got confused by the claim that a vaccine with an efficacy of 95% prevents 95% of vaccinated individuals exposed to the virus from developing the disease (44.8% of correct answers). 63.7% of the respondents incorrectly assessed that a vaccine with an efficacy of 95% prevents the development of a severe form of the disease in 95% of cases, and 53.2% failed to identify that it reduces the incidence rate of the disease by 95% among vaccinated individuals. Regarding knowledge and confidence in knowledge about the notion of p-value, a similar pattern of responses could be observed throughout most of the claims, characterized by a small majority of correct answers under high confidence, a medium peak of low-confidence uncertain responses and a lower peak of incorrect answers under high confidence. As such, 52.5% of the respondents correctly identified that the p-value returned by a statistical test represents the probability of observing a similar or greater effect under the null hypothesis (H0) and 71.4% that it is dependent on the tested sample size. Overall, participants correctly identified that it does not represent the size of the observed difference between the groups (61.1% of correct answers), it does not establish the existence of a necessarily noteworthy effect (59.1% of correct answers) and it does not represent the probability that the tested hypothesis (H1) is true (64.6% of correct answers). A different profile of responses was observed towards the confusing claim that the p-value allows to conclude that the observed effect really exists if its value is lower than the alpha comparison threshold (fixed arbitrarily, most often at 5%): indeed, the correct answers rate for this *hard claim* was of about 28.1% of all respondents.

Comparison between vaccine efficacy and p-value exercises. The number of correct answers and the confidence judgments of participants in the two first exercises were compared using paired t-tests and the Pearson's correlation coefficient (r). Overall, the accuracy and

confidence were slightly lower for the p-value than for the vaccine efficacy exercise, resulting in a higher distance to the correct answer (mean = 39.2, sd = 18.0 and mean = 34.2, sd = 14.6, respectively; paired t-test, t = -6.4, df = 755, p = 2.3e-10). The mean number of correct answers was 3.36 (sd = 1.50) over 6 claims for “p-value” exercice and 3.85 (sd = 1.18) for “vaccine efficacy” exercise (paired t-test, t = 7.98, df = 755, p = 5.4e-15) ; participants’ reported mean confidence (from 0 to 100) was 72.9 (sd = 23.3) for “p-value” exercice and 79.5 (sd = 18.9) for “vaccine efficacy” exercise (paired t-test, t = 8.13, df = 755, p = 1.7e-15). Later analysis further confirmed the slightly although significantly lower confidence in the “p-value” exercise previously described (Wald $\chi2$ = 68.2, df = 1, p < 10e-5, for more details, see *Supplementary Table 2A*).

Linear mixed-effects model (lme4 R package v1.1-21)<br>and Type II Wald chi-square tests (car R package v3.0-7)			
**variable**	**Chisq**	**Df**	**Pr(>Chisq)**
correctness	823.1	1	***<0.0001***
performance	181.5	12	***<0.0001***
profile	0.2	2	0.88
exercise	68.2	1	***<0.0001***
gender	0.5	1	0.48
research	0.3	1	0.56
correctness × performance	97.4	10	***<0.0001***
correctness × profile	2.9	2	0.24
performance × profile	18.3	21	0.63
correctness × performance × profile	36.9	18	***0.005***

A : **Main and interaction effects**

Pairwise comparisons using Tukey’s method (emmeans R package v1.4.5)					
**contrast**	**performance**	**estimate**	**standard error**	**z ratio**	**p-value**
correct - incorrect	0				
	1	12.2	19.6	0.6	0.53

	2	50.6	7.3	7.0	*<0.0001*
	3	32.5	4.5	7.3	*<0.0001*
	4	31.3	2.6	12.1	*<0.0001*
	5	26.6	1.8	14.5	*<0.0001*
	6	20.1	1.5	13.0	*<0.0001*
	7	17.0	1.4	12.0	*<0.0001*
	8	14.2	1.4	9.8	*<0.0001*
	9	11.7	1.7	6.8	*<0.0001*
	10	14.8	2.5	5.9	*<0.0001*
	11	31.5	6.7	4.7	*<0.0001*
	12				

B : **Variation of sensitivity with performance**

Pairwise comparisons using Tukey's method (emmeans R package v1.4.5)						
**contrast**	**performance**	**profile**	**estimate**	**standard error**	**z ratio**	**p-value**
correct - incorrect	1	Student	15.5	27.6	0.6	0.57
		Resident				
		Physician	8.9	27.6	0.3	0.75
	2	Student	63.7	10.2	6.2	*<0.0001*
		Resident	58.0	20.4	2.8	*0.0046*
		Physician	28.4	11.8	2.4	*0.016*
	3	Student	40.0	5.3	7.5	*<0.0001*
		Resident				
		Physician	11.2	7.9	1.4	0.15
	4	Student	29.8	3.1	9.7	*<0.0001*
		Resident	39.9	9.3	4.3	*<0.0001*
		Physician	27.5	5.1	5.4	*<0.0001*
	5	Student	24.2	2.3	10.6	*<0.0001*
		Resident	23.0	4.9	4.7	*<0.0001*
		Physician	28.1	3.5	7.9	*<0.0001*
	6	Student	17.8	1.9	9.5	*<0.0001*
		Resident	18.9	3.9	4.8	*<0.0001*
		Physician	21.4	3.1	6.9	*<0.0001*
	7	Student	14.4	1.6	8.8	*<0.0001*
		Resident	19.3	3.1	6.2	*<0.0001*
		Physician	18.1	3.6	5.0	*<0.0001*
	8	Student	14.2	1.8	8.1	*<0.0001*
		Resident	15.1	3.0	5.1	*<0.0001*
		Physician	9.6	3.4	2.8	*0.0055*

	9	Student	8.8	2.2	3.9	***<0.0001***
		Resident	8.5	3.8	2.3	***0.023***
		Physician	20.1	3.5	5.7	***<0.0001***
	10	Student	12.1	3.7	3.3	***0.0010***
		Resident	9.8	5.7	1.7	0.08
		Physician	21.1	4.3	4.9	***<0.0001***
	11	Student	14.5	9.2	1.6	0.12
		Resident	46.8	19.5	2.4	***0.016***
		Physician	48.3	11.3	4.3	***<0.0001***

C : **Variation of sensitivity with profile**

Supplementary Table 2. Tests based on linear mixed-effects model for "vaccine efficacy" and "p-value" exercises 1 & 2

Results of the Type II Wald chi-square tests (A) on the confidence, with factors: correctness (2 levels), performance (12 levels), participants' profile (3 levels) and covariates: exercise (2 levels), gender (2 levels) and research time (2 levels). (B) Difference of confidence between correct and incorrect answers (discrimination, a measure of metacognitive sensitivity) according to performance. (C) Difference of confidence between correct and incorrect answers according to performance and profile. P-values and adjusted p-values less than 0.05 are highlighted by ***italic bold font***. Of note, no resident scored 1 nor 3/12.

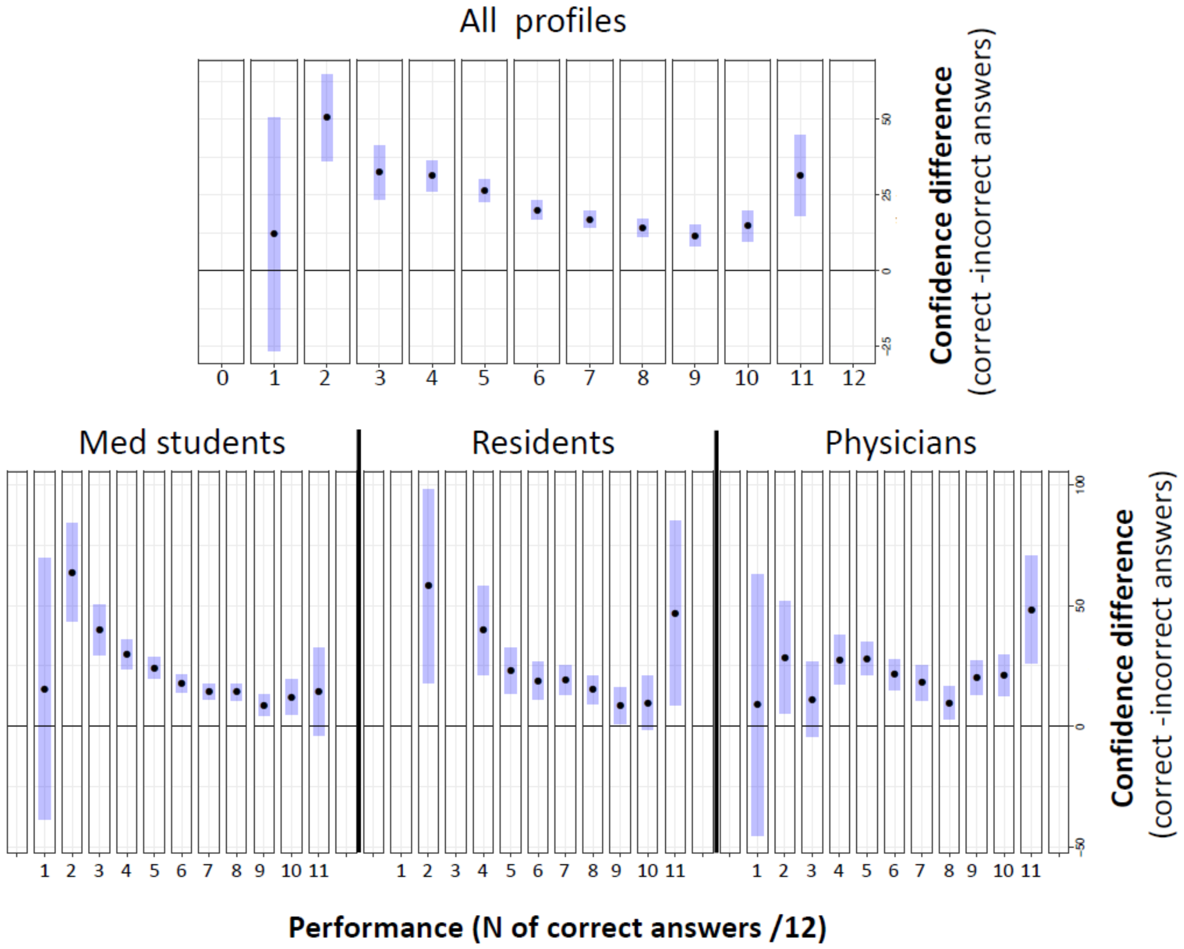

<u>Supplementary Figure 3</u>. Confidence difference (correct - incorrect answers) according to performance and participant's profiles (mean and SEM)

The observed variation of discrimination across performance (triphasic fluctuation of discrimination while performance increases with a maximum at performance = 2/12) appears stronger among medical students compared to physicians, and to a lesser extent among residents compared to physicians (retested three way interaction with 2 levels for participant's profile factor : medical students *vs* physicians: Wald $\chi 2 = 31.0$, df = 10, p = 0.0006; residents *vs* physicians: Wald $\chi 2 = 12.0$, df = 8, p = 0.15; medical students *vs* residents: Wald $\chi 2 = 4.6$, df = 8, p = 0.79). Of note, no resident scored 1 nor 3/12.

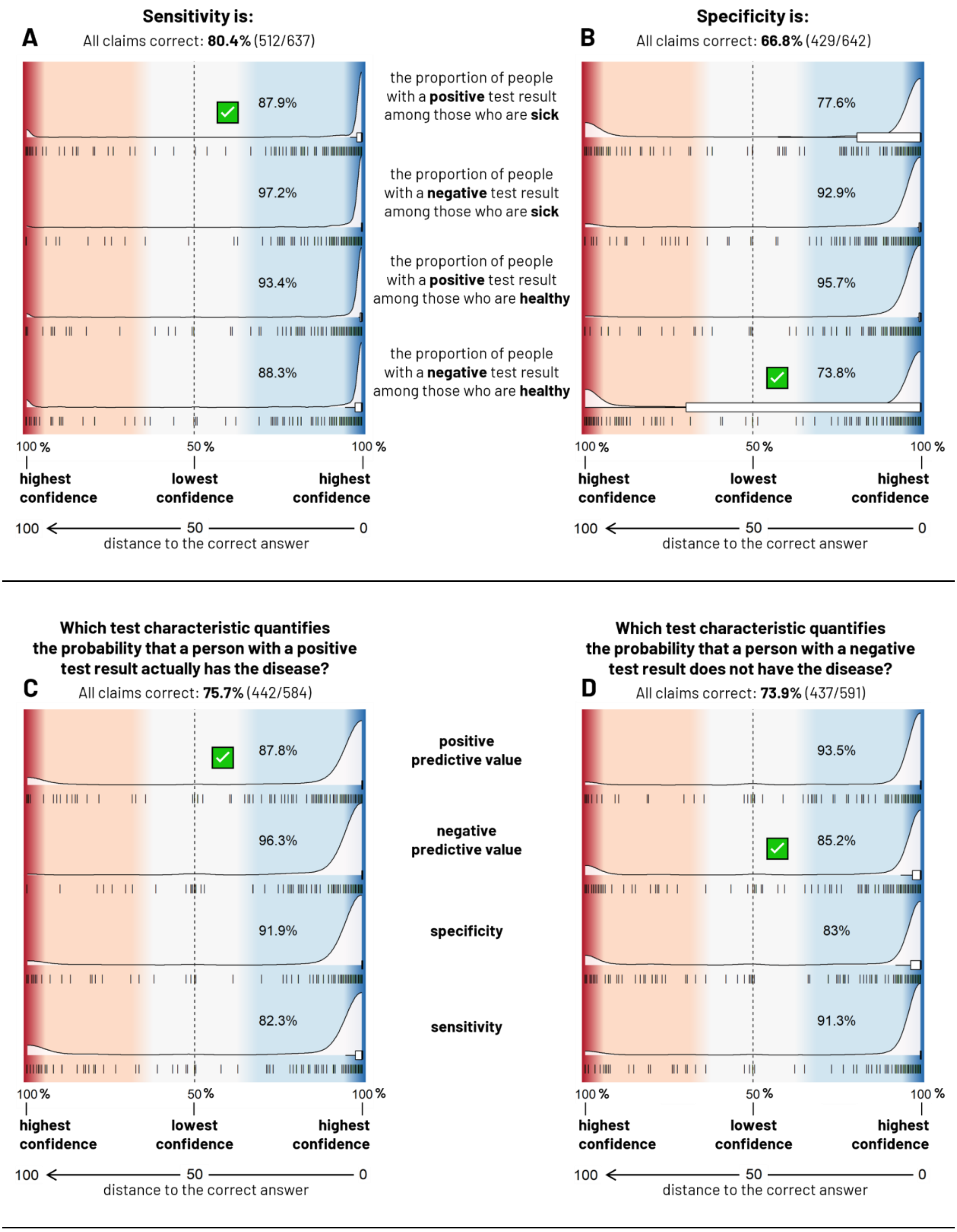
Sensitivity is:
A
All claims correct: 80.4% (512/637)
87.9%
97.2%
93.4%
88.3%
the proportion of people with a positive test result among those who are sick
the proportion of people with a negative test result among those who are sick
the proportion of people with a positive test result among those who are healthy
the proportion of people with a negative test result among those who are healthy
Specificity is:
B
All claims correct: 66.8% (429/642)
77.6%
92.9%
95.7%
73.8%
100 %
50 %
100 %
highest confidence
lowest confidence
highest confidence
100
50
0
distance to the correct answer
Which test characteristic quantifies the probability that a person with a positive test result actually has the disease?
C
All claims correct: 75.7% (442/584)
87.8%
96.3%
91.9%
82.3%
positive predictive value
negative predictive value
specificity
sensitivity
Which test characteristic quantifies the probability that a person with a negative test result does not have the disease?
D
All claims correct: 73.9% (437/591)
93.5%
85.2%
83%
91.3%

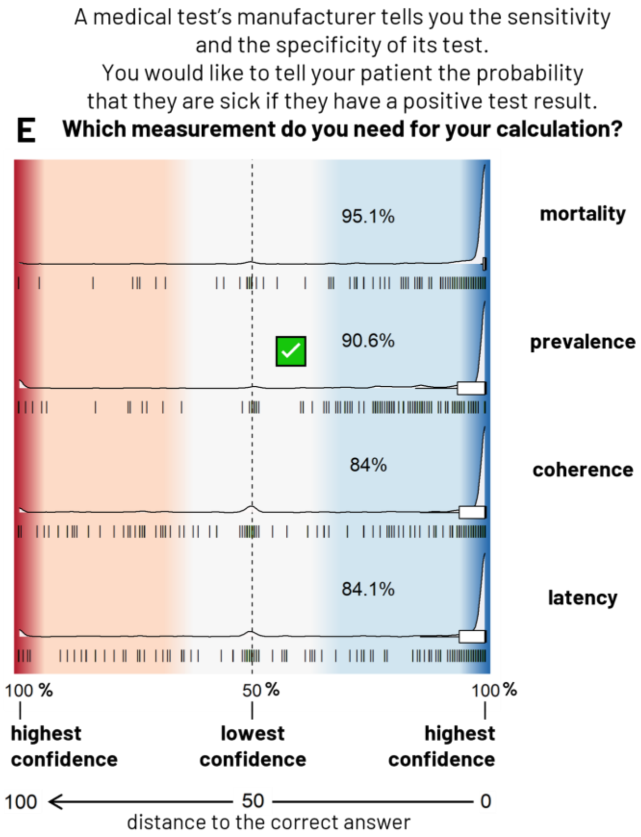

Supplementary Figure 4. **Responses to the theoretical questions of "test results interpretation" exercise**

The density and box plots represent the distribution of participants' responses for each of the 6 proposed claims. The collected data are here mapped onto a double-sided probability scale ranging from 50% (lowest confidence, the participant answered randomly) to 100% (maximal confidence judgment), both for correct (blue area) and incorrect (red area) answers. Also represented on the x-axis, the distance to the correct answer (d) is defined as a score composed both by the accuracy and confidence, ranging from 0: correct answer with maximal confidence to 100: incorrect answer with maximal confidence through 50: "I do not know", $d < 50$ and $d > 50$ respectively corresponding to correct and incorrect answers. Each vertical line stands for a response. The percentage of this exercise's participants who gave a correct answer is indicated in each blue area. True claims are indicated by a green ticked box.

Multiple linear regression model and<br>Type II ANOVA F-tests (car R package v3.0-7)				
variable	Sum of Squares	Df	F value	Pr(>F)
correctness	583.9	3	29.47	**<0.0001**
framing	26.7	1	4.04	**0.045**
correctness × framing	12.9	3	0.65	0.58
Residuals	4445.1	673		

A : Main and interaction effects

Estimated marginal means of confidence by framing<br>(emmeans R package v1.4.5)					
framing	response	standard error	df	lower confidence limit	upper confidence limit
CP	82,0	1,2	676	79,6	84,4
NF	85,1	1,0	676	83,1	87,2

B : Estimated marginal means of confidence by framing

Pairwise comparisons using Tukey's method (emmeans R package v1.4.5)					
contrast	estimate	standard error	df	t ratio	p-value
Incorrect - Incorrect sensitivity	13.1	2.2	676	6.0	***<0.0001***
Incorrect - Incorrect specificity	22.7	2.3	676	9.7	***<0.0001***
Incorrect - Correct	15.5	2.6	676	5.9	***<0.0001***
Incorrect sensitivity - Incorrect specificity	9.5	2.0	676	4.8	***<0.0001***
Incorrect sensitivity - Correct	2.4	2.3	676	1.0	0.72
Incorrect specificity - Correct	-7.1	2.4	676	-2.9	***0.019***

C : Variation of confidence according to correctness

Supplementary Table 3. Tests based on multiple linear regression model for exercise 3 (PPV calculation task)

Results of the Type II ANOVA F-tests (A) on confidence, with the following factors: correctness (4 levels), and framing (2 levels). For this analysis, correctness had 4 levels because we differentiated incorrect from confusion with sensitivity/specificity (thus also incorrect) responses. (B) Based on estimated marginal means (i.e. group means estimated by the model) with standard error (SE), confidence judgments for answers given with the NF framing were slightly greater than with the CP framing (85.1 ± 1.0 vs 82.0 ± 1.2, respectively) (C) Confidence in incorrect responses attributable to confusions was found to be higher than confidence in other incorrect responses, and rather similar (or higher) to confidence in correct answers. P-values and adjusted p-values less than 0.05 are highlighted by ***italic bold font***.

ANNEX

► Phase 1

- **“vaccine efficacy” exercise**

"Analyses regarding messenger RNA vaccines demonstrate approximately 95% efficacy in reducing the number of symptomatic, virologically confirmed COVID-19 cases among people aged 18 or above older with no serologic or virologic evidence of prior SARS-VoV-2 infection and who received both injections."

“Les analyses concernant les vaccins à ARN messager mettent en évidence une efficacité d’environ 95% sur la réduction du nombre de cas de COVID-19 symptomatiques, virologiquement confirmés, chez les sujets de 18 ans et plus, sans preuve sérologique ou virologique d’une infection antérieure au SRAS-VoV-2 et ayant reçu les 2 injections.”

Haute Autorité de Santé. Stratégie de vaccination contre le SARS-CoV-2 – Place du vaccin à ARNm COMIRNATY® (BNT162b2). Saint-Denis La Plaine: HAS; 2020.
&
Haute Autorité de Santé. Stratégie de vaccination contre la Covid-19 – Place du Vaccin Moderna Covid-19 mRNA (nucleoside modified) dans la stratégie. Saint-Denis La Plaine: HAS; 2021.

A vaccine against coronavirus with an efficacy of 95%:
- prevents the development of a severe form of the disease in 95% of cases
- prevents 95% of vaccinated individuals exposed to the virus from developing the disease
- reduces the incidence rate of the disease by 95% among vaccinated individuals ✅
- prevents a single vaccinated individual from developing the disease for 95% of his exposures to the virus
- has been evaluated in a clinical trial to collect the data necessary to calculate its efficacy ✅
- is 95% safe: has only a 5% risk of inducing side effects

- **“p-value” exercise**

In the scope of clinical trials, when we compare the efficacy of 2 treatments, statistical tests are used in order to evaluate the level of significance of observed differences between the 2 groups.

Dans le cadre des essais cliniques, lorsqu'on compare l'efficacité de 2 traitements, des tests statistiques sont utilisés pour évaluer le niveau de significativité des différences observées entre les 2 groupes.

The p-value returned by a statistical test:
- allows to conclude that the observed effect really exists if its value is lower than the alpha comparison threshold (fixed arbitrarily, most often at 5%)
- represents the probability of observing a similar or greater effect if the null hypothesis (H0) were true ✅
- establishes the existence of a necessarily noteworthy effect

- represents the importance of the observed difference between the groups
- represents the probability that the tested hypothesis (H1) is true
- is dependent on the tested sample size ✅

- **"test results interpretation" exercise**

  - **pre-calculation theoretical questions:**

A diagnosis test entails 2 important parameters: its sensitivity and its specificity.

Sensitivity is:
- the proportion of people with a positive test result among those who are sick ✅
- the proportion of people with a negative test result among those who are sick
- the proportion of people with a positive test result among those who are healthy
- the proportion of people with a negative test result among those who are healthy

A diagnosis test entails 2 important parameters: its sensitivity and its specificity.

Specificity is:
- the proportion of people with a positive test result among those who are sick
- the proportion of people with a negative test result among those who are sick
- the proportion of people with a positive test result among those who are healthy
- the proportion of people with a negative test result among those who are healthy ✅

  - **PPV calculation task:**

"Analysis of antigenic detection tests of the SARS-CoV-2 virus suggest
a sensitivity of 90% and a specificity of 99%."

Haute Autorité de Santé. Revue rapide sur les tests de détection antigénique du virus SARS-CoV-2. Saint-Denis La Plaine: HAS; 2020.

  - **conditional probabilities**

You prescribe a Covid-19 test for a patient, having in mind the following information:

- in the population to which the patient belongs, the disease prevalence (the probability that a person has the disease) is 0,4%,
- the test sensitivity (the probability that the test is positive if a person has the disease) is 90%,
- the test specificity (the probability that the test is negative if a person does not have the disease) is 99%.

  - **natural frequencies**

You prescribe a Covid-19 test for a patient, having in mind the following information:

- in the population to which the patient belongs, 40 persons out of 10 000 have the disease,
- 36 out of these 40 patients will receive a positive test result,
- 9 860 out of 9 960 healthy persons will receive a negative test result.

  - **question:**

If the test result is positive, what is the probability that the tested patient truly has Covid-19?

- **post-calculation theoretical questions:**

Which test characteristic quantifies the probability that a person with a positive test result actually has the disease?

- positive predictive value ✅
- negative predictive value
- specificity
- sensitivity

Which test characteristic quantifies the probability that a person with a negative test result does not have the disease?

- positive predictive value
- negative predictive value ✅
- specificity
- sensitivity

A medical test's manufacturer tells you the sensitivity and the specificity of its test. You would like to tell your patient the probability that they are sick if they have a positive test result. Which measurement do you need for your calculation?

- mortality
- prevalence ✅
- coherence
- latency

- **Explanations**
  - CP

► In order to answer this question, it is necessary to calculate the **positive predictive value** using *conditional probabilities*, and more specifically the Bayes theorem:

$$P(A|B) = \frac{P(B|A)P(A)}{P(B)}$$

so:

$$P(A/B) = \frac{P(A) * P(B/A)}{P(A) * P(B/A) + P(\bar{A}) * P(B/\bar{A})}$$

In our case, we need to calculate the probability that the patient really does have the disease knowing that he received a positive test result, so:

$$P(malade \mid +) = \frac{P(malade) * P(+ \mid malade)}{P(malade) * P(+ \mid malade) + P(saine) * P(+ \mid saine)}$$

According to our data:

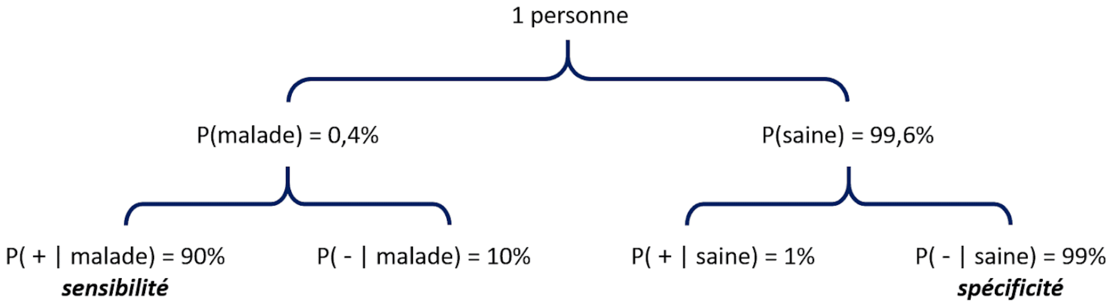

so:

$$P(malade \mid +) = \frac{0{,}004 * 0{,}9}{0{,}004 * 0{,}9 + 0{,}996 * 0{,}01} \simeq 0{,}26 \simeq 26\%$$

► According to the calculation, the probability that the patient actually has Covid-19 if he received a positive antigenic test result is about 26%.

- NF

► In order to answer this question, it is necessary to calculate the **positive predictive value** that represents the probability that the patient really does have the disease, knowing that he received a positive test result. According to our data:

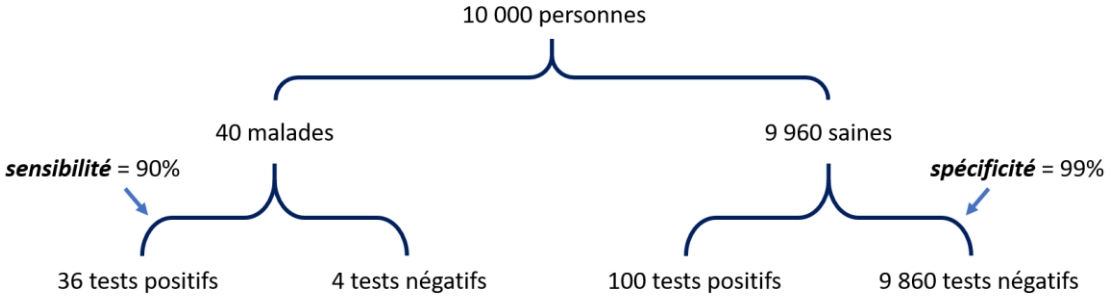

So:

$$P(malade \mid +) = \frac{36}{36 + 100} \simeq 0{,}26 \simeq 26\%$$

► According to the calculation, the probability that the patient actually has Covid-19 if he received a positive antigenic test result is about 26%.

► Phase 2

Framings A and B give the exact same information.

You prescribe to a patient a detection test for a disease D, having in mind the following information::

Framing A

- in the population to which the patient belongs, the disease prevalence (the probability that a person has the disease) is 1%,
- the test sensitivity (the probability that the test is positive if a person has the disease) is 90%,
- the test specificity (the probability that the test is negative if a person does not have the disease) is 91%.

Framing B

- in the population to which the patient belongs, 10 persons out of 1 000 have the disease,
- 9 out of these 10 patients will receive a positive test result,
- 901 out of 990 healthy persons will receive a negative test result.

If the test result is positive, what is the probability that the tested patient truly has disease D?
